# Bi-steric mTORC1 inhibitors induce apoptotic cell death in tumor models with hyperactivated mTORC1

**DOI:** 10.1172/JCI167861

**Published:** 2023-11-01

**Authors:** Heng Du, Yu Chi Yang, Heng-Jia Liu, Min Yuan, John M. Asara, Kwok-Kin Wong, Elizabeth P. Henske, Mallika Singh, David J. Kwiatkowski

**Affiliations:** 1 Division of Pulmonary and Critical Care Medicine, Brigham and Women’s Hospital, Boston, Massachusetts, USA.; 2 Department of Biology, Revolution Medicines Inc., Redwood City, California, USA.; 3 Division of Signal Transduction, Beth Israel Deaconess Medical Center, Boston, Massachusetts, USA.; 4 Department of Medicine, Harvard Medical School, Boston, Massachusetts, USA.; 5 Laura and Isaac Perlmutter Cancer Center, New York University Langone Medical Center, New York, New York, USA.; 6 Division of Hematology and Medical Oncology, Department of Medicine, Laura and Isaac Perlmutter Cancer Center, NYU Grossman School of Medicine, New York University Langone Health, New York, New York, USA.

**Keywords:** Oncology, Therapeutics, Cancer

## Abstract

The PI3K/AKT/mTOR pathway is commonly dysregulated in cancer. Rapalogs exhibit modest clinical benefit, likely owing to their lack of effects on 4EBP1. We hypothesized that bi-steric mTORC1-selective inhibitors would have greater potential for clinical benefit than rapalogs in tumors with mTORC1 dysfunction. We assessed this hypothesis in tumor models with high mTORC1 activity both in vitro and in vivo. Bi-steric inhibitors had strong growth inhibition, eliminated phosphorylated 4EBP1, and induced more apoptosis than rapamycin or MLN0128. Multiomics analysis showed extensive effects of the bi-steric inhibitors in comparison with rapamycin. De novo purine synthesis was selectively inhibited by bi-sterics through reduction in JUN and its downstream target PRPS1 and appeared to be the cause of apoptosis. Hence, bi-steric mTORC1-selective inhibitors are a therapeutic strategy to treat tumors driven by mTORC1 hyperactivation.

## Graphical abstract

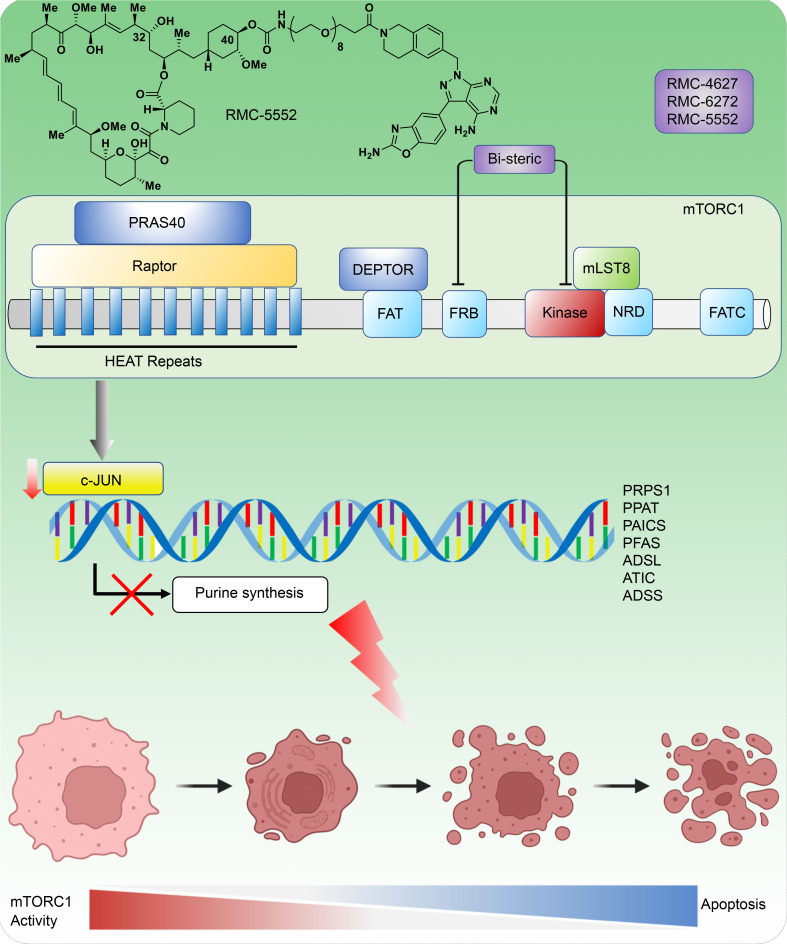

## Introduction

The PI3K/AKT/mTOR network is a critical intracellular signaling pathway directing cell growth and metabolism in physiological and pathological conditions (1). The evolutionarily conserved mammalian target of rapamycin (mTOR) is a serine/threonine kinase ubiquitously expressed in mammalian cells (2). mTOR is the key protein in mTORC1 and mTORC2 protein complexes (3), and mTORC1 regulates a signaling cascade involved in protein synthesis, gene expression, glucose and lipid metabolism, and nucleotide biosynthesis (4).

mTORC1 phosphorylates 4E-binding protein 1 (4EBP1) and S6 kinase (S6K), downstream targets involved in cap-dependent translation initiation and elongation (5). Complete loss of *TSC1* or *TSC2*, occurring in both tuberous sclerosis complex (TSC) tumors and a variety of cancer types (6–8), leads to constitutive unregulated activation of mTORC1 (9).

Given the frequent activation of PI3K/AKT/mTOR signaling in human tumors, several generations of mTOR inhibitors have been developed (1). Rapalogs have been approved by the FDA for the treatment of several TSC-associated tumors as well as renal cell carcinoma (RCC) (10). However, rapalogs have shown limited benefit in treating patients with RCC, bladder cancer (BLCA), and cancers of any origin with biallelic mutations in either *TSC1* or *TSC2* (11–13), which may be due to the incomplete suppression of mTORC1 kinase activity by rapalogs, including a lack of effect on p-4EBP1^T37,46^, p-PRAS40^S183^, and p-Grb10^S150^ (14). MLN0128 (sapanisertib), a second-generation ATP-competitive inhibitor, inhibits all the mTORC1 substrates, but also inhibits mTORC2, likely contributing to reported toxicities necessitating lowered dosage that may limit its clinical efficacy (15).

Third-generation “bi-steric” mTORC1-selective inhibitors have been developed, consisting of a rapamycin derivative and an mTOR active-site inhibitor connected by a linker region (16). These bi-steric inhibitors overcome the limitations of the first- and second-generation inhibitors by virtue of being mTORC1 selective and able to inhibit p-4EBP1. We hypothesized that these potent mTORC1-selective inhibitors have the potential for greater benefit than rapalogs in the treatment of tumors with mTORC1 hyperactivation. We show that bi-steric inhibitors, RMC-4627, RMC-6272, and RMC-5552 (a clinical candidate) (17), drive rapid and durable suppression of p-S6K^T389^, p-4EBP1^T37,46^, p-mTOR^S2481,S2448^, and p-PRAS40^S183,T246^. Bi-steric inhibitors are tolerated and have consistent antitumor activity both in vitro and in vivo. Furthermore, these bi-steric mTORC1-selective inhibitors induce global reprogramming of anabolism and catabolism as compared with rapamycin, indicating broad effects of more effective mTORC1 inhibition. Notably, RMC-5552 is currently undergoing clinical evaluation in patients with cancer (ClinicalTrials.gov NCT04774952). Our work provides evidence that bi-steric mTORC1 inhibitors provide improved therapeutic benefit as compared with the currently used rapalogs in tumors with high mTORC1 activity, due to important differences in the effects of these agents on purine metabolism. These studies provide strong support for human clinical trials of selective bi-steric mTORC1 inhibitors in patients with TSC1/TSC2-deficient cancers.

## Results

### The PI3K/AKT/mTOR pathway is widely dysregulated in cancer, and the evolutionarily conserved mTORC1 is a central therapeutic target.

Genetic alterations involving the PI3K/AKT/mTOR pathway are common in cancer, with 4,689 of 10,800 (43%) cancers from The Cancer Genome Atlas (TCGA) showing an alteration in 1 or more of the following genes: *PIK3CA* (17%, mainly mutation and amplification), *PTEN* (12%, mainly deletion), *PIK3R1* (4%, mainly mutation), *AKT1* (2.2%, mainly amplification), *RPTOR* (4%, mainly amplification), *TSC2* and *TSC1* (3%, 2.7%, mainly mutation), and *MTOR* (4%, mainly amplification) (Supplemental Figure 1A; supplemental material available online with this article; https://doi.org/10.1172/JCI167861DS1). Patients with cancers with an alteration in this pathway show worse overall survival outcomes than those without (Supplemental Figure 1B). In addition, reverse-phase protein assay (18) data from 7,663 TCGA patient samples from 31 tumor types demonstrated that p-4EBP1 and mTOR activity, but not p-S6K activity, was associated with poor prognosis (Supplemental Figure 1, C–E), indicating the importance of 4EBP1 dysregulation in tumor progression (19, 20).

### Bi-steric mTORC1-selective inhibitors potently suppress tumor cell proliferation.

Two bi-steric mTORC1 inhibitors (RMC-4627 and RMC-6272) were assessed on multiple tumor cell lines with mTORC1 hyperactivation secondary to *TSC1* or *TSC2* loss. Mutation in one or both of these genes is seen in perivascular epithelioid cell tumors (PEComa) (50%) (21), BLCA (8%–10%) (22), RCC (4%–7%) (23), hepatocellular carcinoma (HCC) (3–5%) (24), and lung adenocarcinoma (LUAD) (~1%) (25).

**Figure 1:**
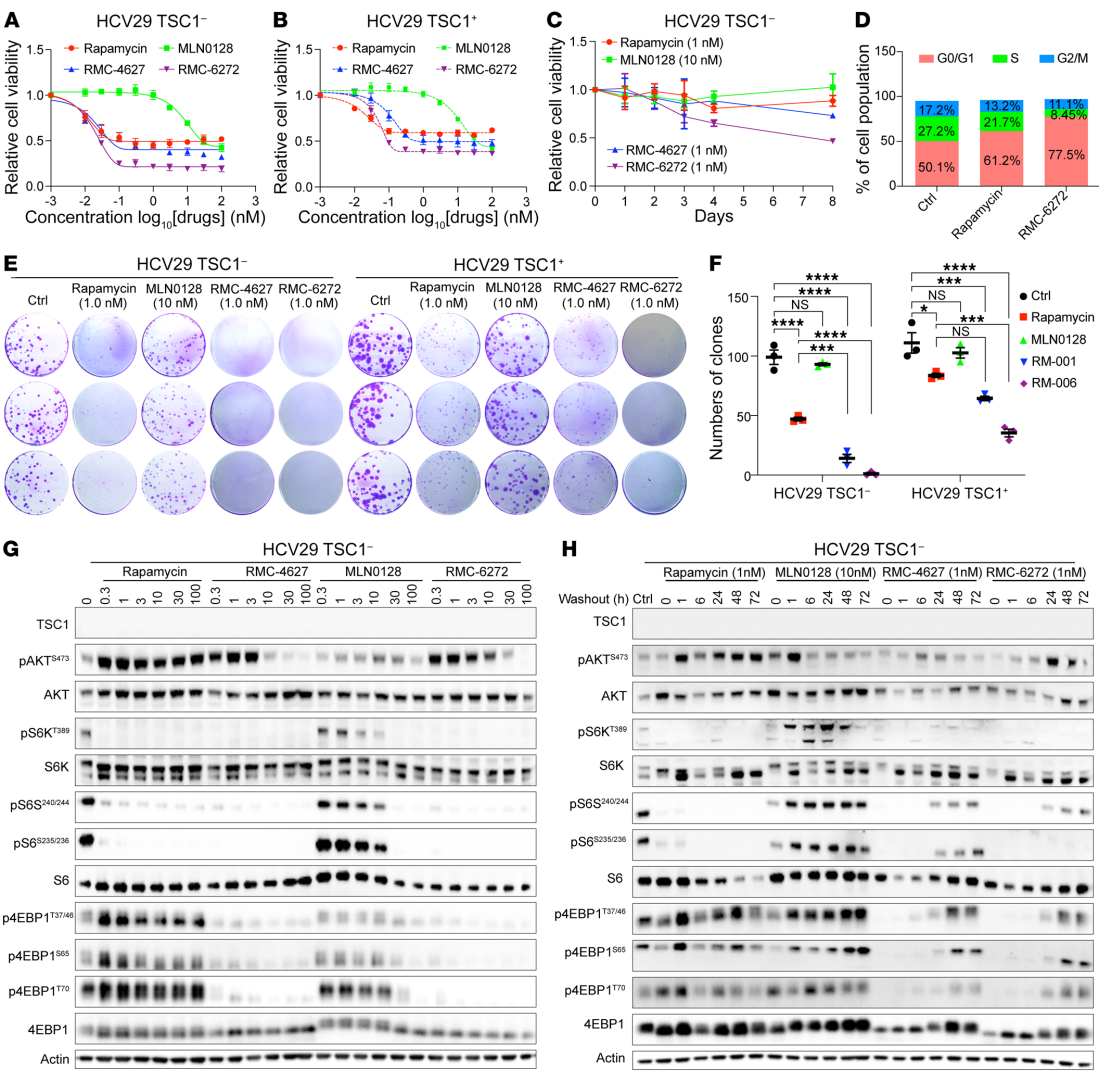
Bi-steric mTOR inhibitors show potent inhibition of tumor cell proliferation. (**A** and **B**) Growth inhibition curves of TSC1-null HCV29 cells (**A**) and TSC1–add-back HCV29 cells (**B**) treated with rapamycin, MLN0128, RMC-4627, and RMC-6272. Each dot and error bar on the curves represent mean ± SD (*n* = 6). (**C**) Cell proliferation rate of HCV29 TSC1-null cells treated with rapamycin (1 nM), MLN0128 (10 nM), RMC-4627 (1 nM), and RMC-6272 (1 nM) for 24 hours followed by washout. Each dot and error bar on the curves represent mean ± SD (*n* =6). (**D**) Cell cycle analysis of HCV29 TSC1-null cells treated with rapamycin (1 nM) or RMC-6272 (1 nM) for 24 hours. (**E** and **F**) Long-term low-dilution clonogenic growth assay of TSC1-null HCV29 (**E**, left) and TSC1–add-back HCV29 (**E**, right) cells, and quantification of clone numbers (**F**). Each bar is the median of *n* = 3 measurements. One-way ANOVA was used. *P < 0.05, ***P < 0.001, ****P < 0.0001. (**G**) The effect of rapamycin, MLN0128, RMC-4627, and RMC-6272 on mTORC1 signaling in HCV29 TSC1-null cells treated with different concentrations (nM) of inhibitors for 4 hours. (**H**) The effect of rapamycin, MLN0128, RMC-4627, and RMC-6272 on mTORC1 signaling in HCV29 TSC1-null cells treated as in **C** for 24 hours followed by washout.

TSC1-null HCV29 (BLCA cell line) and its TSC1–add-back derivative were treated with rapamycin, MLN0128, and bi-steric mTORC1 inhibitors. Both RMC-4627 and RMC-6272 showed a maximal 70% inhibition of growth compared with rapamycin (maximum 50%) and MLN0128 (55% at 100 μM; Figure 1A). TSC1-expressing HCV29 cells had an approximately 5-fold higher IC_50_ than TSC1-null HCV29 cells (Figure 1, A and B, and Supplemental Table 1). Similar findings were seen in another 13 pairs of TSC-null and TSC–add-back/wild-type cell lines from different tumor types (Supplemental Table 1), including TSC1-null BLCA cells (Supplemental Figure 2, A and B), TSC2-null angiomyolipoma cells (Supplemental Figure 2C), TSC2-null HCC cells (Supplemental Figure 2, D and E), Tsc1- and Tsc2-null mouse embryonic fibroblasts (Supplemental Figure 2, F and G), Tsc2-null mouse RCC cells (Supplemental Figure 2, H, I, and M), and Tsc1-null mouse LUAD cells (Supplemental Figure 2, J–L). Cell proliferation was more durably blocked by the bi-steric inhibitors (7 days) than by rapamycin (2–3 days) and MLN0128 (1–2 days), with RMC-6272 causing the strongest and most durable inhibition (Figure 1C and Supplemental Figure 2, N–Q).

Low-dilution clone formation decreased up to 50% with rapamycin and showed little effect with MLN0128, while the bi-steric inhibitors caused at least 70% reduction in multiple TSC-null cell lines, including several with more than 95% reduction (Figure 1, E and F, and Supplemental Figure 3). Both bi-steric inhibitors caused a milder decrease in growth of the TSC1–add-back/control cells, consistent with a selective effect. RMC-6272 caused a more dramatic reduction in clone formation than RMC-4627 for all cell lines. RMC-6272 (1 nM, 24 hours) also led to G_0_/G_1_ cell cycle arrest in contrast to rapamycin (1 nM, 24 hours) (Figure 1D).

### Bi-steric inhibitors cause rapid and complete mTORC1 inactivation.

Both bi-steric inhibitors at 0.3 nM almost completely abolished mTORC1 activity, as shown by profound reduction in p-S6K^T389^, p-S6^S235,236^, p-S6^S240,244^, p-4EBP1^T37,46^, p-4EBP1^S65^, and p-4EBP1^T70^ levels (Figure 1G and Supplemental Figure 4, A–P). The reduction in p-S6K^T389^, p-S6^S235,236^, and p-S6^S240,244^ was also seen with rapamycin at 0.3–1 nM; in contrast, rapamycin had no effect on p-4EBP1^T37,46^, p-4EBP1^S65^, and p-4EBP1^T70^ even at 100 nM. MLN0128 reduced levels of all of these phospho-sites at doses of 10–30 nM in all cell lines. Immunoblot analysis of p-AKT^S473^ was used to assess the activity of mTORC2. Rapamycin and the 2 bi-steric inhibitors showed some increase in p-AKT^S473^ at lower concentrations, consistent with feedback activation of PI3K signaling that follows mTORC1 inhibition, while MLN0128 inhibited p-AKT^S473^ at 100 nM (Figure 1G and Supplemental Figure 4, A–P). The bi-steric inhibitors showed durable inhibition of phosphorylation of S6K, S6, and 4EBP1 at all sites for more than 24 hours. Rapamycin durably blocked p-S6K^S389^, p-S6^S235,236^, and p-S6^S240,244^ only. In MLN0128-treated cells, phosphorylation recovery began 1 hour after washout (Figure 1H and Supplemental Figure 4, Q–T).

### Integrative multiomics analysis reveals differential global reprogramming induced by RMC-6272 in comparison with rapamycin.

To gain a detailed view of how bi-steric mTORC1-selective inhibition contrasted with the effects of rapamycin, multiomics analysis (transcriptomic, metabolomic, lipidomic, proteomic, phosphoproteomic) was performed on 2 representative cell lines (HCV29 TSC1-null and 705 Tsc2-null) treated with RMC-6272 (3 nM, 24 hours), rapamycin (10 nM, 24 hours), or DMSO as the negative control.

**Figure 2:**
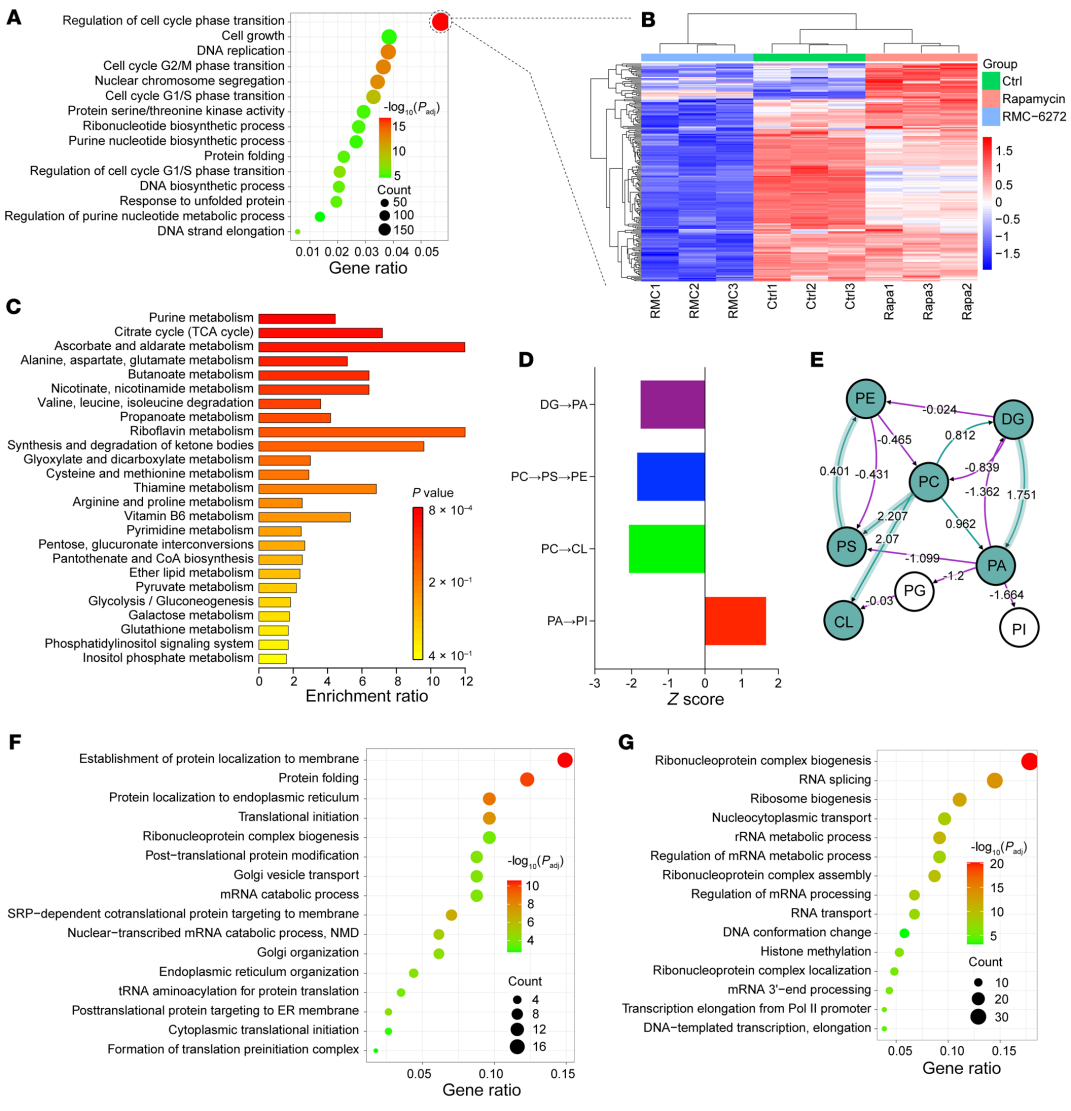
Multiomics analysis of effects of RMC-6272 versus rapamycin. (**A**) Gene set enrichment analysis (GSEA) of RNA data comparing RMC-6272– and rapamycin-treated TSC1-null HCV29 cells. The most downregulated pathways after RMC-6272 treatment are shown. (**B**) Seventy-three cell cycle genes were decreased in mRNA expression (FPKM) in RMC-6272–treated HCV29 cells compared with DMSO- or rapamycin-treated cells. (**C**) Purine metabolism was the metabolic pathway showing the most significant decrease in metabolite levels following RMC-6272 compared with rapamycin treatment in HCV29 TSC1-null cells, by MSEA. (**D** and **E**) Lipid map indicating lipid changes comparing RMC-6272– with rapamycin-treated HCV29 TSC1-null cells. Green nodes correspond to active lipids and arrows to conversion pathways. *Z* scores were used to assess significance. Reactions with a positive *z* score have green arrows, while negative *z* scores are colored purple. (**F**) Global proteomics analysis of RMC-6272– versus rapamycin-treated HCV29 TSC1-null cells led to identification of proteins involved in protein localization, targeting, and folding as being most downregulated with RMC-6272 treatment compared with rapamycin. (**G**) Global phosphoproteomics analysis of RMC-6272– versus rapamycin-treated 705 Tsc2-null cells identified ribonucleoprotein biogenesis and RNA splicing pathways enriched for phosphoproteins that were downregulated with RMC-6272 treatment compared with rapamycin using GSEA.

RMC-6272 treatment led to changes in mRNA expression compared with rapamycin or DMSO (Supplemental Figure 5, A and D). Pathway enrichment analysis showed that genes downregulated by RMC-6272 versus rapamycin were enriched for those involved in cell cycle/cell phase transition and DNA replication in both HCV29 TSC1-null and 705 Tsc2-null cell lines (Figure 2, A and B, Supplemental Figure 5, B and C, and Supplemental Tables 2 and 3). These findings were consistent with the cell cycle arrest induced by RMC-6272 (Figure 1D), and suggest the importance of inhibition of 4EBP1 phosphorylation to prevent cell proliferation induced by mTORC1 (26).

Three hundred polar metabolites were analyzed using mass spectrometry. Metabolite set enrichment analysis (MSEA) identified purine metabolism as the metabolite set that was most enriched for metabolites decreased by RMC-6272 in comparison with rapamycin for both human HCV29 TSC1-null (Figure 2C) and mouse 705 Tsc2-null (Supplemental Figure 7A) cell lines. This differential effect of RMC-6272 on purine metabolites in comparison with rapamycin was further verified using another four TSC1/TSC2-null cell lines (Supplemental Figure 6), for which differential effects on purine metabolite levels was among the top-ranked metabolite sets in each cell line.

MSEA showed no major change in glycolysis (Supplemental Figure 11A). Both rapamycin and RMC-6272 caused a significant reduction of glucose uptake in comparison with DMSO-treated cells, while RMC-6272 showed a greater effect than rapamycin in 4 of 4 cell lines (Supplemental Figure 11B).

Lipidomic analysis showed that both rapamycin and RMC-6272 caused perturbations among the 43 different subtypes of lipids detected. As shown by lipid maps, RMC-6272 led to an apparent conversion from phosphatidic acid to phosphatidylinositol in both cell lines (Figure 2, D and E, and Supplemental Figure 5, F and G), indicating a differential effect on lipid biogenesis in comparison with rapamycin. Lipid subtypes (ceramides [Cer], phosphatidylinositol [PI], and triglycerides [TG]), fatty acid length, and number of unsaturated bonds did not show a significant difference between rapamycin and RMC-6272 treatment (Supplemental Figure 12).

Global proteomics analysis also showed major differences in the effects of the bi-steric mTORC1-selective inhibitor RMC-6272 in comparison with rapamycin. Proteins downregulated by RMC-6272 treatment relative to rapamycin were enriched for translation and ribosome-related proteins according to pathway analysis (adjusted *P* value < 0.05, |log_2_(fold change)| > 1.5; Figure 2F and Supplemental Figure 5E), consistent with effects of non-phosphorylated 4EBP1 on synthesis of those proteins induced by RMC-6272. In addition, there was little overlap between the most downregulated mRNAs and the most downregulated proteins, consistent with effects of 4EBP1 on translation initiation.

Since mTORC1 phosphorylates multiple targets and initiates a downstream phosphorylation cascade, global phosphoproteomics analysis was performed on Tsc2-null 705 cells treated with rapamycin, RMC-6272, and DMSO as control. Both rapamycin and RMC-6272 caused dephosphorylation at multiple sites on S6 (Supplemental Figure 5J); in contrast, only RMC-6272 eliminated phosphorylation at multiple sites of 4EBP1 (Supplemental Figure 5I). These findings are consistent with immunoblot findings (Figure 1G and Supplemental Figure 4).

Differential analysis of the effects of RMC-6272 versus rapamycin on the phosphoproteome using Kyoto Encyclopedia of Genes and Genomes (KEGG) pathways indicated that proteins involved in ribonucleoprotein biogenesis, RNA splicing, and ribosome biogenesis were the proteins whose phosphorylation was most commonly downregulated (Figure 2G). Genes undergoing alternative splicing (AS) identified by RNA sequencing (RNA-Seq) were then analyzed. There were 400 genes that had a difference in AS induced by RMC-6272 versus rapamycin and were involved in the DNA metabolic process and other pathways (Supplemental Figure 5H).

Although both rapamycin and RMC-6272 decreased protein synthesis, RMC-6272 had a much stronger effect with 80% reduction in protein synthesis in comparison with 50% for rapamycin (Supplemental Figure 11, D and E).

Autophagy level was detected by LC3, p62, and p-ULK1. Both rapamycin and RMC-6272 induced autophagy. mTOR suppresses autophagy partly by phosphorylating ULK1 at Ser757 (27). p-ULK1^S757^ is insensitive to rapamycin (14), but very sensitive to RMC-6272. Hence, RMC-6272 enhanced autophagy to a greater extent than rapamycin, as judged by p62 and LC3 expression (Supplemental Figure 11C).

### Bi-steric mTORC1-selective inhibition suppresses de novo purine synthesis through the mTORC1/JUN/PRPS1 axis.

**Figure 3:**
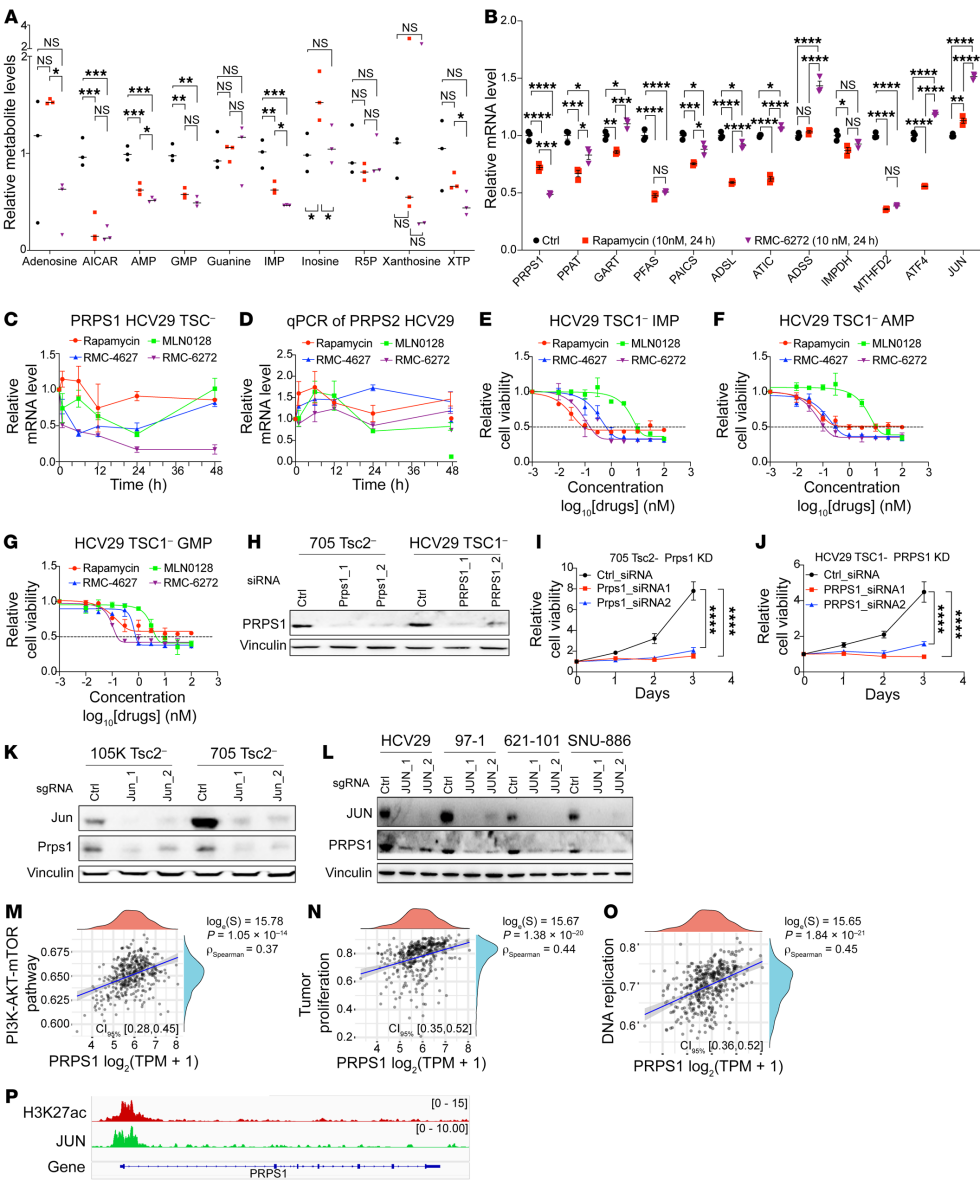
De novo purine synthesis is suppressed by RMC-6272 in an mTORC1/JUN/PRPS1-dependent manner. (**A**) Purine metabolites following RMC-6272 (3 nM, 24 hours) or rapamycin (3 nM, 24 hours) treatment of HCV29 TSC1-null cells. (**B**) mRNA levels (RNA-Seq) of de novo purine synthesis enzymes and related TFs in HCV29 TSC1-null cells treated for 24 hours. (**C** and **D**) Quantitative reverse transcriptase PCR assessment of PRPS1 (**C**) and PRPS2 (**D**) mRNA levels in HCV29 cells treated with rapamycin (10 nM), MLN0128 (10 nM), RMC-4627 (3 nM), or RMC-6272 (3 nM) for different times. (**E**–**G**) IC_50_ curves of HCV29 cells supplied with 100 nM of IMP (**E**), AMP (**F**), or GMP (**G**) and treated with rapamycin, MLN0128, RMC-4627, and RMC-6272. (**H**) Immunoblot analysis of PRPS1 KD in 705 and HCV29 cell lines. (**I** and **J**) Cell proliferation assay after knockdown of PRPS1 in 705 and HCV29 cells. (**K**) mRNA levels of Prps1 and Prps2 in Jun CRISPR KO 105K and 705 cells. (**L**) PRPS1 mRNA expression is decreased after JUN CRISPR KO in multiple cell lines. (**M**–**O**) Correlation between mRNA expression level of PRPS1 and the PI3K/AKT/mTOR pathway (**M**), tumor cell proliferation (**N**), and DNA replication (**O**) in TCGA data. (**P**) H3K27ac and JUN CUT&RUN data for the 621-101 cell line show open chromatin with JUN binding near the transcriptional start site of PRPS1. **A** and **B**: Dots are independent measurements, and lines are the median (*n* = 3). **C**–**G**, **I**, and **J**: Each dot and error bar represent mean ± SD (*n* = 3). One-way ANOVA was used. *P < 0.05, **P < 0.01, ***P < 0.001, ****P < 0.0001.

Multiple purine metabolites were decreased to a greater extent in RMC-6272–treated HCV29 cells in comparison with rapamycin treatment, including adenosine, AICAR, AMP, IMP, and GMP (Figure 3A), with similar findings in 4 other TSC1/TSC2-null cell lines (Supplemental Figure 6). Notably, there was no significant change in ribose 5-phosphate after RMC-6272 treatment (Figure 3A and Supplemental Figure 6, B, D, F, and H), suggesting that downregulation of the purine de novo synthesis pathway was the cause of these changes. PRPS1, which catalyzes the first and rate-limiting step of purine de novo synthesis, was reduced at both mRNA (Figure 3B and Supplemental Figure 7C) and protein levels after 24 hours of either RMC-4627 or RMC-6272 at 20 nM (Supplemental Figure 4, P, U, and V). Quantitative reverse transcriptase PCR analysis confirmed that PRPS1, but not PRPS2, which performs a similar catalytic function, was decreased up to 90% by RMC-6272 treatment compared with rapamycin, which caused a much smaller decrease (~20%) (Figure 3, C and D, and Supplemental Figure 7, D and E). Purine nucleotides are needed for many intracellular processes, including DNA replication and cell proliferation, and a lack of purine nucleotides would be expected to cause cell cycle arrest and effects on expression of genes regulating cell cycle progression, as noted in our RNA-Seq analysis (Figure 2A and Supplemental Figure 5B). To examine the hypothesis that purine nucleotide deficiency explained the growth-inhibitory effects of bi-steric inhibitors, we performed an add-back experiment with exogenous purines. The addition of IMP, AMP, and GMP at 100 nM each to the culture medium increased the IC_50_ of bi-steric inhibitors 4- to 10-fold on the TSC1-null HCV29 cell line and the Tsc2-null 705 cell line (Figure 3, E–G, and Supplemental Figure 7, H–J; compare with Figure 1A, Supplemental Figure 2I, and Supplemental Table 1). There was a minor effect of the addition of these purines on the IC_50_ of rapamycin and no effect on the IC_50_ of MLN0128. Furthermore, PRPS1 knockdown (KD) by siRNA had a significant effect on HCV29 cell proliferation in comparison with control siRNA-treated cells (Figure 3, H–J). In addition, Prps1 knockout (KO) by CRISPR/Cas9 induced cell apoptosis with no surviving cells when applied to each of 3 different murine Tsc1/Tsc2-null cell lines (Supplemental Figure 7G), confirming the critical role of Prps1 in purine synthesis for cell growth and survival. Interestingly, PRPS1 mRNA expression is associated with reduced survival and positively correlated with DNA replication, tumor proliferation, and the PI3K/AKT/mTOR signature in the TCGA data set (Figure 3, M–O), confirming its broad essential role in cell growth and proliferation.

Having discovered that PRPS1 mRNA levels are decreased after mTORC1 suppression, we sought to determine the mechanism of this effect. JASPAR (28) was used to predict transcription factors (TFs) that bind to the PRPS1 promoter region, and several candidate TFs that may regulate PRPS1 expression were identified, including JUN, E2F4, JUND, and ATF4. mTORC1 inhibitors had little effect on ATF4 expression at 24 hours (Supplemental Figure 4, P, U, and V), and no effect on E2F4 and JUND (data not shown). In contrast, JUN protein levels decreased dramatically starting at 3 hours of bi-steric inhibitor treatment, whereas rapamycin had a milder effect on JUN expression beginning at 24 hours (Supplemental Figure 4P). RNA-Seq data, in contrast, showed an approximately 50% increase in JUN mRNA in both HCV29 and 705 cells in response to RMC-6272 treatment (Figure 3B and Supplemental Figure 7B). This is consistent with prior reports that JUN autoregulates its expression through an inhibitory binding interaction with its own promoter region, such that a decrease in JUN protein triggers upregulation of its mRNA (29). The decreased JUN expression upon mTORC1 inhibition suggests that JUN may be activated in some manner downstream of mTORC1. TCGA pan-cancer reverse-phase protein assay data showed that JUN mRNA level is positively correlated with mTORC1 activity in 27 of 32 cancer types (data not shown). To further explore the mechanism of how mTORC1 regulates JUN, cells were treated with actinomycin D (ActD). ActD reduced JUN protein levels at 12 hours, independent of DMSO/rapamycin/RMC-6272 treatment. In contrast, cycloheximide (CHX) treatment caused a rapid and near-complete loss of JUN protein only with concurrent bi-steric treatment (Supplemental Figure 8, E and F). The ActD effect suggests that JUN protein expression requires continued RNA synthesis, while the CHX effect only with bi-steric treatment suggests that complete mTORC1 inhibition caused a reduction in JUN protein stability.

To examine the relationship between JUN and PRPS1 in greater detail, JUN CRISPR gene deletion was performed in 6 different TSC1/TSC2-null cell lines. Both JUN and PRPS1 protein levels were reduced (Figure 3, K and L). To determine whether JUN was regulating PRPS1 expression directly, JUN and H3K27ac CUT&RUN was performed. JUN bound to PRPS1 in a representative cell line with mTORC1 hyperactivation (621-101), indicating that PRPS1 was a direct transcriptional target (Figure 3P). Our results were further validated by publicly available JUN ChIP-Seq data on 2 cancer cell lines (MDA-MB-231 and A549) (30, 31) (Supplemental Figure 8M). Furthermore, MSEA showed that the purine synthesis pathway was the most decreased pathway in c-JUN^KD^ cells in comparison with c-JUN^WT^ cell lines (Supplemental Figure 8, A and B).

Twenty-eight of 32 TCGA cancer types had increased expression of both JUN and PRPS1 in comparison with their respective normal tissues, indicating the importance of JUN and PRPS1 in tumorigenesis and tumor progression. In summary, these findings indicate that mTORC1 regulates purine de novo synthesis through modulation of JUN expression and downstream PRPS1.

### mTORC1 bi-steric inhibitors are more effective than rapamycin in multiple TSC-deficient tumor models with mTORC1 hyperactivation in vivo.

**Figure 4:**
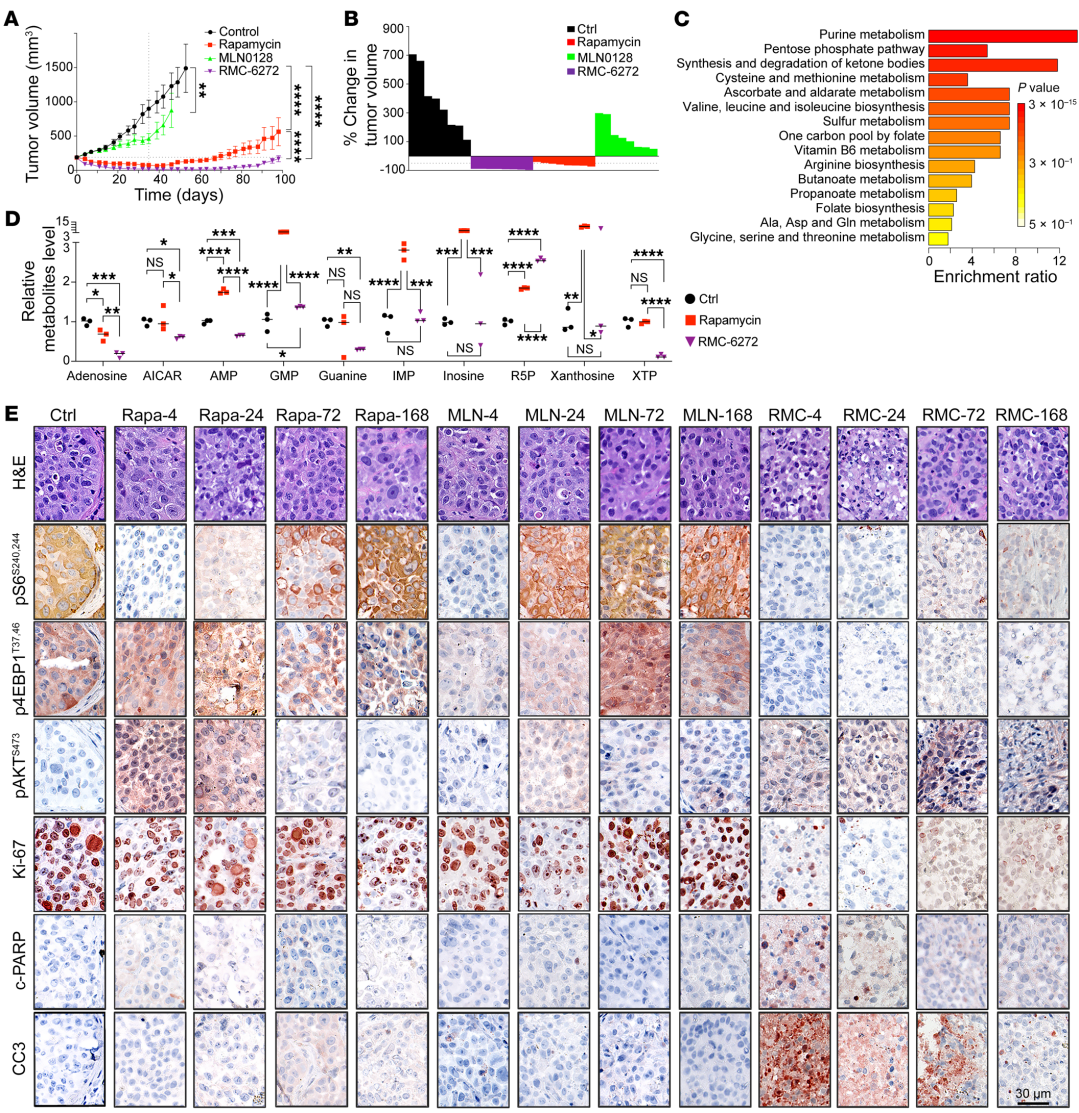
Bi-steric compounds showed greater tumor suppression in vivo than rapamycin. (**A**) Tumor volume of human BLCA PDX-2211 treated for 4 weeks followed by 2-month treatment cessation. Each dot and error bar represent mean ± SD (*n* = 8 mice per group, 2 tumors per mouse). Rapamycin: 3 mg/kg, 3 times/wk. MLN0128: 0.75 mg/kg, 5 times/wk. RMC-6272: 8 mg/kg, once per week. Student’s *t* test was used. **P < 0.01, ****P < 0.0001. (**B**) Waterfall plot showing the response of tumors in different treatment groups after 4-week treatment (as in **A**). Each bar represents a mouse with 2 tumors (*n* = 8 mice per group). (**C** and **D**) Purine metabolites are those most decreased by global metabolite analysis of a human BLCA PDX after 24 hours of treatment with RMC-6272 (8 mg/kg) compared with rapamycin (3 mg/kg). Dots are individual values (*n*=3); a median line is shown. One-way ANOVA was used. *P < 0.05, **P < 0.01, ***P < 0.001, ****P < 0.0001. (**E**) H&E and IHC staining of BLCA PDX dosed once (same dose as in **A**) followed by washout for 4, 24, 72, and 168 hours as indicated at the top. Scale bar: 30 μm. c-PARP, cleaved PARP; CC3, cleaved caspase 3.

**Figure 5:**
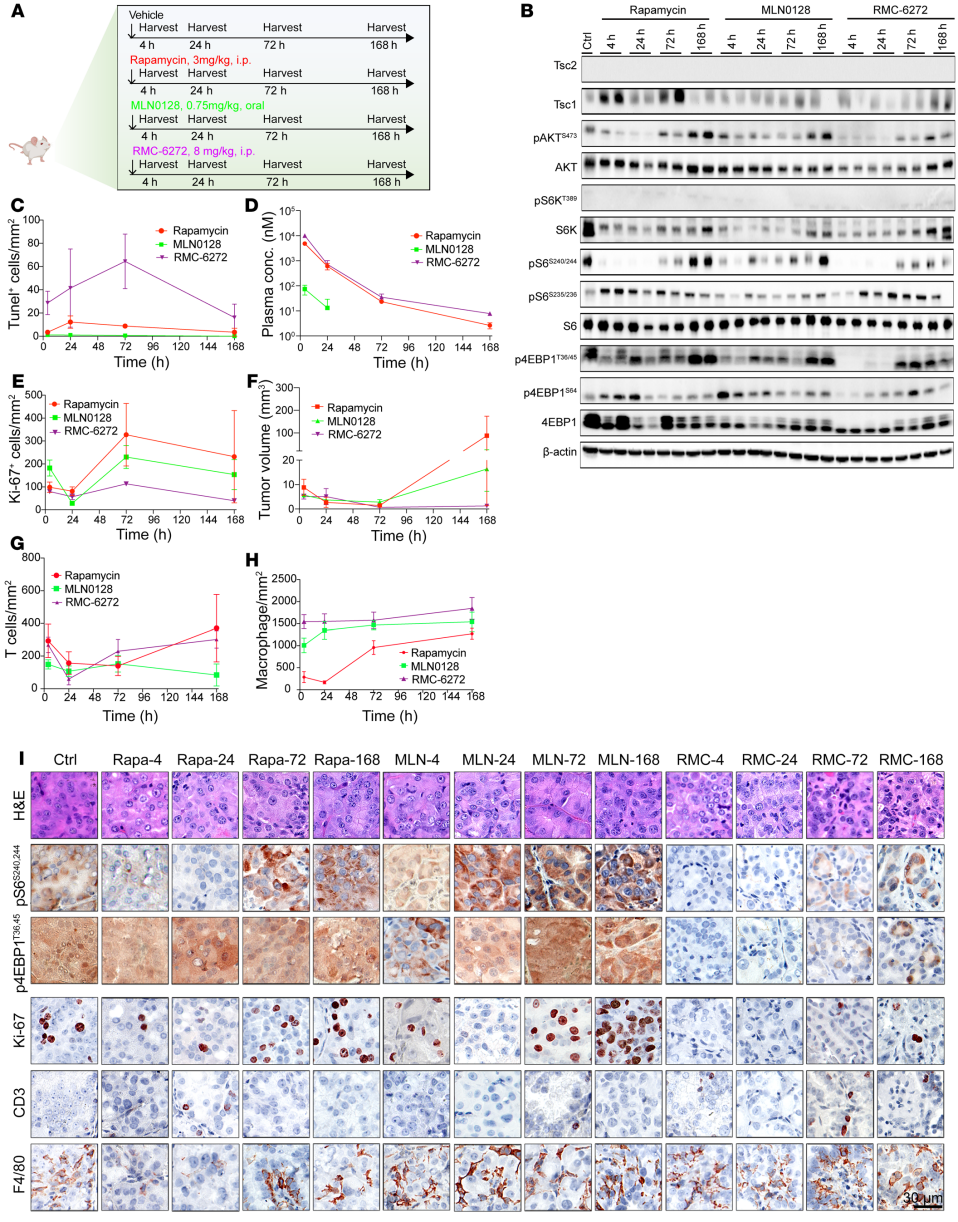
Single-dose assessment of bi-steric inhibitors in the *Tsc2^+/–^ A/J* mouse kidney cancer model. (**A**) Schematic diagram of the single-dose treatment strategy. (**B**) Immunoblotting of mTORC1 signaling pathway in whole kidneys after single dose, harvested at different time points. (**C**) Quantification of tumor cell apoptosis marker TUNEL. (**D**) Plasma concentrations of different compounds after 1 dose. (**E**) Quantification of proliferation marker Ki-67. (**F**) Measurement of kidney tumor volume in each mouse kidney. Different tumor sizes reflected the treatment effect and individual variation. (**G** and **H**) T cell (CD3) and macrophage (F4/80) infiltration. CD3 was used as a pan–T cell marker. F4/80 was used as a macrophage marker. (**I**) H&E and IHC staining of kidney tumors. For **C**–**H**, each bar and error bar represent mean ± SD (*n* = 6)

Consistent with our mechanistic observations, RMC-6272 drove deeper tumor regression in 4 weeks of treatment, and significantly delayed tumor regrowth upon treatment cessation, compared with rapamycin, both at translatable doses, in a human TSC1-null BLCA patient-derived xenograft (PDX) model (*n* = 8 per group; Figure 4, A and B). MLN0128 at maximum tolerated dose showed little activity in this model. Modest weight loss occurred after each dose of RMC-6272, followed by recovery (Supplemental Figure 9A). To examine the in vivo mechanism of tumor response, a separate cohort of mice with PDX received a single dose of RMC-6272, followed by sacrifice at different time points (Figure 5A). Both immunoblot and IHC showed that S6K and 4EBP1 phosphorylation sites were eliminated by RMC-6272 for a week. In contrast, these markers showed a more limited and transient response to rapamycin and MLN0128 (Figure 4E and Supplemental Figure 10B). RMC-6272 treatment also led to dephosphorylation of multiple mTORC1 components, including mTOR, Raptor, and PRAS40 (Supplemental Figure 10B). Strikingly, RMC-6272–treated PDX tumors showed near-complete ablation of Ki-67 staining and evidence of apoptosis as indicated by c-PARP and cleaved caspase-3 (CCS3) staining. These were not seen with either rapamycin or MLN0128 treatment (Figure 4E and Supplemental Figure 9K). Metabolite analysis of the tumors at 24 hours (Figure 4, C and D) but not 4 hours (Supplemental Figure 9, B and C) showed a profound decrease in purine metabolites, in comparison with rapamycin, with adenosine showing a 90% reduction. JUN IHC also showed that total and nuclear JUN levels were reduced by RMC-6272 at all times, while this was more limited and transient with rapamycin and not seen at all with MLN0128 (Supplemental Figure 9, L and M). PRPS1, but not PRPS2, was decreased at the mRNA level (Supplemental Figure 9, I and J).

We evaluated further the in vivo antitumor activity of bi-steric mTORC1-selective inhibitors by testing RMC-5552 in 3 human BLCA PDX models with TSC1/TSC2 deficiency, including the same model in which RMC-6272 was assessed. RMC-5552 is a clinical candidate representing the bi-steric mTORC1-selective inhibitor class, and currently being tested in a phase I clinical trial (NCT04774952). All 3 BLCA PDX models with TSC1/TSC2 deficiency were sensitive to RMC-5552, which caused significant tumor volume reduction in 2 of 3 PDX models, and delayed tumor growth in the third (Supplemental Figure 9, D–H).

**Figure 6:**
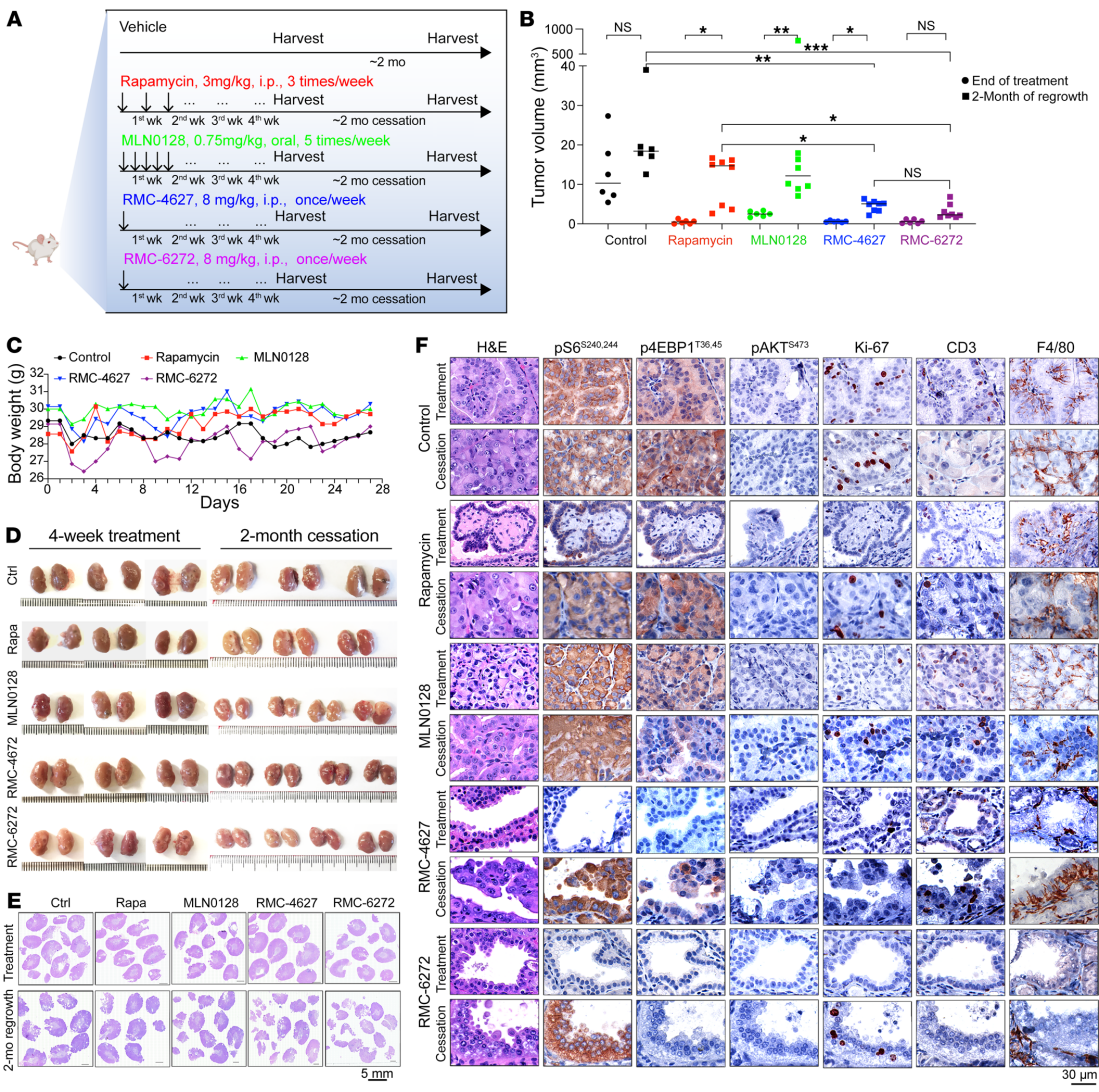
Bi-steric compounds showed more dramatic tumor suppression and less tumor regrowth in the *Tsc2^+/–^* A/J mouse kidney cancer model. (**A**) Schematic diagram of treatment strategy. (**B**) Tumor volume from semiquantitative analysis of H&E slides immediately after treatment or 2 months after treatment. Dots are individual values (*n* = 6); a median line is shown. One-way ANOVA was used. *P < 0.05, **P < 0.01, ***P < 0.001. (**C**) Toxicity evaluation of compounds as judged by body weight. Each dot represents median (*n* = 6). (**D** and **E**) Images of whole mouse kidneys (**D**) and H&E-stained kidney sections (**E**) after 4 weeks of treatment and another 2-month tumor regrowth from mice as in **A**, both immediately after treatment and after 2-month regrowth. Scale bar: Mm ruler (**D**) and 5 mm (**E**). (**F**) H&E and IHC staining of kidney sections after 4 weeks of treatment and after 2 months of tumor regrowth. Scale bar: 30 μm.

**Figure 7:**
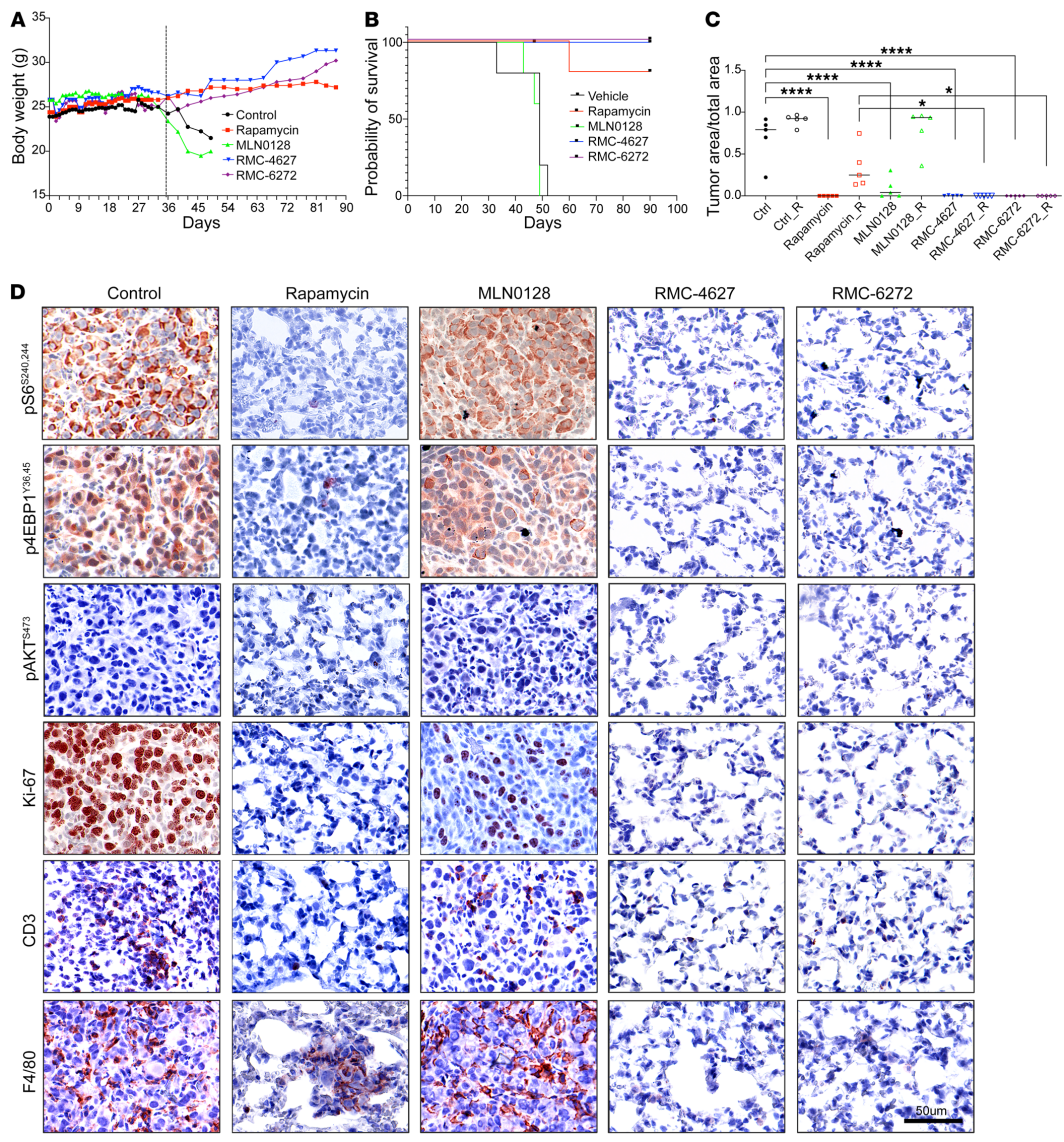
Lung tumors were eliminated after 4-week bi-steric inhibitor treatment in a mouse lung tumor model generated by tail vein injection of TTJ (Tsc2-null) kidney cancer cells. (**A**) Body weight of mice during treatment (4 weeks) and treatment cessation (up to 2 months after stopping treatment). Each dot represents median (*n* = 5). (**B**) Survival of mice with different treatments. Median survivals for vehicle, rapamycin, MLN0128, RMC-4627, and RMC-6272 were 49, >90, 49, >90, and >90 days, respectively. (**C**) Tumor size quantification of lung tumors in different treatment groups. “_R” indicates 2 months regrowth. Dots are individual values (*n* = 5); a median line is shown. One-way ANOVA was used. *P < 0.05, ****P < 0.0001. (**D**) IHC staining of mouse lungs after 4 weeks of treatment, for each of 5 treatments. Scale bar: 50 μm.

We also examined the effects of the bi-steric inhibitors on a genetically engineered mouse model of Tsc2 RCC (*Tsc2^+/–^* A/J mice; Figure 6A) and a mouse lung tumor model (Figure 7). Both rapamycin and bi-steric inhibitors were tolerated as assessed by body weight (Figure 6C and Figure 7A) and significantly decreased tumor burden (Figure 6, B, D, and E). RMC-6272 showed less tumor regrowth than rapamycin or MLN0128 after treatment cessation (Figure 6B), particularly in the mouse lung tumor model, in which no tumor regrowth was observed (Figure 7, B–D). Single-dose experiments confirmed that RMC-6272 caused more tumor apoptosis than rapamycin (Figure 5 and Supplemental Figure 10A). RMC-6272 showed a more prolonged suppressive effect on mTORC1 activity as compared with rapamycin (Figure 5, B and I, and Figure 6F), which occurred with reduced tumor cell proliferation and enhanced apoptosis (Figure 5, C–F). Differences in T cell numbers or macrophages were not observed, suggesting that apoptosis was due to intrinsic effects of mTORC1 inhibition on the tumor cells (Figure 5, G and H, and Figure 6F).

## Discussion

The PI3K/AKT/mTOR pathway is commonly dysregulated in human cancer, due to mutation of dominantly acting oncogenes (*PIK3CA*, AKT isoforms, *MTOR*), inactivation of tumor suppressor genes (*PTEN*, *TSC1*, *TSC2*, *PIK3R1*), and amplification of growth-promoting oncogenes (18). All of these events lead to enhanced mTORC1 signaling, which through diverse downstream effectors leads to cell anabolic processes for both critical biochemical intermediates and macromolecules (e.g., ribosomes) that enable cell growth (32). Arguably, the strongest mTORC1-activating genetic events are those in which there is biallelic loss of *TSC1* or *TSC2*. TSC1, TSC2, and TBC1D7 form a protein complex that converts RHEB.GTP to RHEB.GDP, and loss of this TSC protein complex function leads to high levels of RHEB.GTP and constitutive activation of mTORC1 (2).

Several generations of mTORC1 inhibitors have been created. Rapamycin and its analogs, rapalogs (33), cause partial responses in about 10% of RCC patients, and have been reported to have major benefit in occasional cases of bladder cancer and other cancer types with TSC1/TSC2 mutations (13). Rapalogs have shown more consistent benefit for several tumors (renal angiomyolipoma, cortical subependymal giant cell astrocytoma, pulmonary lymphangioleiomyomatosis) that develop as part of the TSC syndrome, as well as sporadically (34, 35). However, rapalogs produce a modest but persistent response in many TSC patients, with an average 50% reduction in tumor volume, and requirement for lifelong treatment since tumors will recur when rapalog therapy is stopped (11).

The rapalog-FKBP12 complex reduces mTORC1 kinase activity in a variable manner on its different substrates, with S6K among the most sensitive, and 4EBP1 among the most resistant, to rapalog inhibition (Figure 1, G and H). There are at least 10 mTORC1 substrates that are insensitive to rapalog inhibition (36). This likely contributes to the limited clinical benefit of rapalog therapy (37).

Bi-steric mTORC1-selective inhibitors were designed to combine the high selectivity of the rapamycin-FKBP12 complex for the FRB domain and an mTOR ATP binding domain moiety, to give high specificity for mTORC1 over mTORC2, along with near-complete inhibition of mTORC1 activity on all of its substrates (38, 39).

RMC-4627 is a bi-steric inhibitor that contains a rapamycin core and PP242-derived active site inhibitor, which are linked through an ether bond at the C40 position on rapamycin and a Peg8 linker. Based on cellular assays using MDA-MB-468 cells, RMC-4627 displays potent inhibition of p-4EBP1 (IC_50_: 1.4 nM) and p-S6K (IC_50_: 0.28 nM) and demonstrates selectivity for mTORC1 over mTORC2 (calculated as p-AKT IC_50_/p-4EBP1 IC_50_), with approximately 13-fold selectivity. RMC-6272 and RMC-5552 are 2 bi-steric inhibitors in which the C40-linked ether chemical handle was exchanged for a C40 carbamate to enable synthetic tractability and incorporated the XL388-derived (RMC-6272) and MLN0128-derived (RMC-5552) active site inhibitors, respectively. RMC-6272 and RMC-5552 display very potent inhibition of p-4EBP1 and p-S6K. Both compounds demonstrate selectivity for mTORC1 over mTORC2, with approximately 27-fold and 40-fold selectivity, respectively. This selectivity is afforded by modulation of both the active site inhibitor and the rapamycin core. For both RMC-6272 and RMC-5552 the carbonyl at the C32 position has been reduced to a hydroxyl group, which serves to modulate binding affinity to FKBP12 and improves chemical stability of the macrocyclic core.

Here we show that several bi-steric mTORC1-selective inhibitors (a) show high selectivity for mTORC1 over mTORC2; (b) completely inhibit mTORC1 activity on all substrates tested; (c) have a higher potency for mTORC1 inhibition in TSC1/TSC2-mutant cancer cell lines as compared with rapamycin, and show modest selectivity (5- to 10-fold shift in IC_50_) for these cells in comparison with parallel or (isogenic) add-back cell lines; (d) show effects in inhibiting the growth of such cell lines in clonal dilution growth assays; and (e) show improvement over rapamycin in the treatment of a variety of in vivo models with mTORC1 hyperactivation due to TSC1/TSC2 loss, including syngeneic xenografts, native mouse genetic models, and human bladder cancer patient-derived xenografts.

Bi-steric mTORC1-selective inhibitors caused global rewiring of cellular metabolism, with significant effects on RNA expression, metabolites, lipids, protein expression, and phosphoprotein levels in comparison with rapamycin. Most importantly, bi-steric inhibitors caused tumor cell apoptosis, in contrast to rapamycin, for several of the tumor models for which superiority to rapamycin was demonstrated.

Our assessment of the omics effects of bi-steric treatment led us to identify a profound reduction in purine metabolites in the bi-steric–treated cells. Subsequent studies identified an axis of transcriptional regulation of purine synthesis downstream of mTORC1, in which JUN drives expression of PRPS1, the critical first enzyme in the de novo purine synthesis pathway. We found that both JUN and PRPS1 are reduced in expression by immunoblotting following the bi-steric inhibitor treatment, both in vitro and in vivo, consistent with the reduction in purine metabolites and constituting potential substrates downstream of mTORC1 inhibition. In addition, several different purines individually rescued the growth-inhibitory effects of the bi-steric inhibitors in vitro, while PRPS1 was shown to be absolutely required for viability of all cell lines tested. Previously an mTORC1/ATF4/MTHFD2 pathway was identified as critical for purine synthesis in cells with mTORC1 activation due to TSC1/TSC2 loss, and sensitive to rapalog inhibition (40). However, we saw no effect on ATF4 expression, and a modest approximately 50% reduction in MTHFD2 expression in response to bi-steric treatment. While it is not completely clear how mTORC1 regulates JUN, we observed that JUN protein levels were decreased by 3 hours after bi-steric inhibitor treatment but not rapamycin treatment, and complete mTORC1 suppression by bi-steric mTORC1-selective inhibitors caused a rapid drop in JUN levels when cotreated with CHX (Supplemental Figure 8E). Thus, mTORC1 appears to regulate JUN translation in a 4EBP1-dependent manner. Further studies are required, including investigation of the possibility that mTORC1 or a downstream kinase phosphorylates JUN, which might regulate its expression level.

In summary, bi-steric mTORC1-selective inhibitors with moieties that bind to both FKBP12 and the ATP binding pocket on mTOR show high specificity for mTORC1, require lower concentrations for mTORC1 inhibition than rapalogs, induce apoptosis in in vivo models, and appear to act (at least in part) by suppressing de novo purine synthesis through reduction in PRPS1 expression. Bi-steric mTORC1-selective inhibitor treatment is also well tolerated in vivo in mouse models at doses that lead to therapeutic benefit. The bi-steric mTORC1-selective inhibitor RMC-5552 is now in human clinical trials and, based on the results presented herein, has the potential to benefit patients with TSC syndrome tumors, and patients with the common cancers in which TSC1/TSC2 mutations drive hyperactivated mTORC1 activity.

## Methods

### Analysis of genetic events (Oncoprint).

Genetic events within the PI3K/AKT/mTOR pathway were downloaded through cBioPortal (https://www.cbioportal.org/oncoprinter).

### mTOR score and prognosis analysis.

The relationship between the p-S6K score (defined as the sum of p-S6K^T389^, p-S6^S235,236^, and p-S6^S240,244^), p-4EBP1 score (defined as the sum of p-4EBP1^T37,46^, p-4EBP1^S65^, and p-4EBP1^T70^), and mTOR score (defined as the sum of p-S6K, p-4EBP1, and p-mTOR^S2448^) and patient prognosis was analyzed as previously described with modifications (18). p-Rictor was not included. Pan-cancer reverse-phase protein assay data, JUN and PRP1 mRNA expression levels, and patient survival data were downloaded from FireBrowse (http://firebrowse.org/).

### Cells and culture conditions.

Human bladder cancer cell lines HCV29, RT4, 97-1, and 639V were described previously (22), and were maintained in DMEM supplemented with 10% FBS and 100 U/mL of penicillin and streptomycin and incubated at 37°C in 5% CO_2_. TSC1–add-back counterpart cell lines for HCV29 and 97-1 were described previously (22). SNU-398 and SNU-886 human hepatocellular carcinoma cell lines were obtained from the Broad Institute and were maintained in RPMI 1640 medium supplemented with 10% FBS and 100 U/mL of penicillin and streptomycin and incubated at 37°C in 5% CO_2_. The TSC2–add-back counterparts of those 2 lines were generated previously (22). Human angiomyolipoma cells (621-101), 105K mouse kidney cyst–adenocarcinoma cells, and their TSC2–add-back counterparts were maintained as previously described (41). Mouse embryo fibroblasts (MEFs) and mouse kidney hybrid oncocytic/chromophobe tumor (HOCT) cells were generated in Dr. Kwiatkowski’s laboratory and maintained as previously described (40). Mouse lung adenocarcinoma cell lines (KTP-267-1B1, KTP-267-2B8, KTP-269-3C4, 857T, 855T, 634T) were generated as previously described (42). TTJ cells and their TSC2–add-back counterpart cells were provided by Vera Krymskaya (43). All the cell lines were tested routinely for mycoplasma contamination, and only mycoplasma-negative cells were used.

### Cell proliferation assay (including washout).

The effect of the compounds on cell proliferation was measured by crystal violet staining. Cells were plated in 96-well plates at a density of 500–2,000 cells (sextuples per condition). The next day, cells were treated with concentrations (0–100 nM) prepared by serial dilution. After 3 days of treatment, cells were fixed by 10% formalin for 10 minutes at room temperature. 0.05% crystal violet solution was added and incubated for 15 minutes at room temperature. Absorbance was read using Synergy HT (BioTek) at 540 nm. The inhibition curves and IC_50_ values were calculated by GraphPad Prism 9.0.

### 14-Day low-dilution clone formation assay.

Long-term effects on cell proliferation were assessed by counting of the number of clones after 200–1,000 cells were seeded on 10 cm plates (triplicates per condition). After 3 days, cells were treated at concentrations selected based on IC_50_ values (with a maximum of 10 nM). Media were changed every 3 days. Cell clones were fixed with 10% formalin and stained with 0.05% crystal violet after 14 days of treatment. The plates were washed extensively and scanned with a flatbed scanner. For quantification, clone numbers with size ≥ 1 mm were counted manually and data analyzed using GraphPad Prism 9.0.

### Cell cycle analysis.

Cell cycle analysis was performed following the manufacturer’s instructions (Abcam, ab-139148). Briefly, cells were harvested and prepared in single-cell suspension after being treated with DMSO, rapamycin, or RMC-6272 for 24 hours. Cell pellets were washed and fixed in 66% ethanol on ice for 2 hours. Cells were then resuspended in propidium iodide plus RNase staining solution at 37°C for 30 minutes. Cell populations were analyzed using FACS (BD LSRFortessa).

### Western blotting.

Cells were washed with cold PBS twice and lysed on ice for 30 minutes in 1× RIPA buffer (Cell Signaling Technology) supplemented with protease and phosphatase inhibitor cocktails (Sigma-Aldrich), followed by centrifugation at 18,000*g* at 4°C. Proteins were extracted from approximately 10 mg tissue samples through bead (Next Advance, SSB05) motion-based homogenizing in 1× RIPA buffer for tumor tissues. Equal amounts (30 μg) of total protein were loaded in NuPAGE gel (Thermo Fisher Scientific), transferred to nitrocellulose membranes (Thermo Fisher Scientific, IB23001), incubated with primary and secondary antibodies, and detected by SuperSignal West Pico PLUS/Femto Maximum Sensitivity chemiluminescent substrate (Thermo Fisher Scientific, 34580, 34095). Primary antibodies against mTOR (2983S), Raptor (2280S), DEPTOR (11816S), mLST8 (3274S), TSC2 (4308S), TSC1 (4906S), AKT (4685S), p-AKT^S473^ (4060S), S6K (2708S), p-S6K^T389^ (9234S), S6 (2217S), p-S6^S235/236^ (4858S), p-S6^S240/244^ (5364S), 4EBP1 (9644S), p-4EBP1^T37/46^ (2855S), p-4EBP1^S65^ (9451S), p-4EBP1^T70^ (9455S), β-actin (3700S), PARP (9532S), cleaved PARP (5625S), caspase-3 (9662S), cleaved caspase-3 (9664S), LC3A/B (4108S), SQSTM1/p62 (88588S), p-ULK1^S757^(6888S), FLAG (14793S), and His (12698S) and anti-rabbit/mouse secondary antibodies were purchased from Cell Signaling Technology (CST). Antibody against FKBP12 (AB92459) was purchased from Abcam. Anti-PPAT antibody (15401-1-AP) was purchased from Proteintech.

Actinomycin D (A9415-5MG; 1 μg/mL) and cycloheximide (C4859-1ML; 10 μM) were from Sigma-Aldrich. MG132 (S2619; 2 μM) was from SelleckChem. ImageJ (NIH) was used to quantify the protein levels using figures generated from Western blots.

### Generation of PRPS1-KO cells.

CRISPR/Cas9–mediated PRPS1-KO cell lines were generated as previously described (44). All-in-one lentiCRISPR vectors were designed and purchased from VectorBuilder. Each all-in-one vector contained 2 different guide RNAs (gRNAs). The gRNA sequences were: *Prps1*_1, 5′-TTCTTATCTTGGCGGGCGTA-3′; *Prps1*_2, 5′-CATTGCAGACCGGCTGAATG-3′; negative control, 5′-GTGTAGTTCGACCATTCGTG-3′, 5′-GTTCAGGATCACGTTACCGC-3′; *PRPS1*_1, 5′-GTCCTCCTGAGGTATGGTAT-3′; *PRPS1*_2, 5′-CGGCTGTCCTAAAGTGGATA-3′; negative control, 5′-GTGTAGTTCGACCATTCGTG-3′, 5′-GTTCAGGATCACGTTACCGC-3′.

### Generation of PRPS1- and JUN-KD cells.

PRPS1 KD was achieved by siRNA transfection. Cells were cultured to 30%–50% confluence in 6-well plates. Transient siRNA transfections were performed using Lipofectamine RNAi/MAX Transfection Reagent (Thermo Fisher Scientific, 13778075) and Opti-MEM (Life Technologies, 31985070) according to the manufacturer’s protocol. Cells were harvested 72 hours after transfection. siRNA oligonucleotides were obtained from Life Technologies (siPRPS1_1, AM16708-111211; siPRPS1_2, AM16708-118242; siPrps1_1, AM16708-152230; siPrps1_2, AM16708-152231; siCtrl, AM4635) and Horizon Discovery Biosciences (ON-TARGETplus Human JUN siRNA, Smartpool, L-003268-00-0010; ON-TARGETplus Human CTRL siRNA, L-005834-00-0005; ON-TARGETplus Mouse Jun siRNA, L-043776-00-0005; ON-TARGETplus Mouse Ctrl siRNA, L-044488-01-0005).

### Quantitative reverse transcriptase PCR of PRPS1, PRPS2, and JUN.

Cells were treated with rapamycin (10 nM), MLN0128 (10 nM), RMC-4627 (1 nM), and RMC-6272 (1 nM) for different times (0, 1, 6, 12, 24, and 48 hours). RNA was extracted using an RNeasy Mini Kit (Qiagen, 74104). cDNA was synthesized from the same amount of RNA using a High-Capacity cDNA Reverse Transcription Kit (Thermo Fisher Scientific, 4368814). The primers used were: PRPS1 (Thermo Fisher Scientific, Hs00751338_s1), PRPS2 (Thermo Fisher Scientific, Hs00267624_m1), Prps1 (Thermo Fisher Scientific, Mm00727494_s1), Prps2 (Thermo Fisher Scientific, Mm00471753_m1), ACTB (Thermo Fisher Scientific, Hs01060665_g1), Actb (Thermo Fisher Scientific, Mm02619580_g1), JUN (Thermo Fisher Scientific, Hs01103582_s1), and Jun (Thermo Fisher Scientific, Mm07296811_s1).

### Measurement of protein synthesis rate.

Protein synthesis rate was tested using Click-iT HPG Alexa Fluor 594 Protein Synthesis Assay Kit (Thermo Fisher Scientific, C10429). Cells were treated with rapamycin (10 nM) or RMC-6272 (10 nM) for 24 hours. Protein synthesis rate was detected by fluorescent microscope or measured by plate reader.

### RNA sequencing.

RNA-Seq was performed at Novogene. Cells were treated with DMSO, rapamycin (10 nM), or RMC-6272 (3 nM) for 24 hours (triplicates per condition). Total RNA was extracted using an RNeasy Mini Kit (Qiagen, 74104). One microgram RNA per sample was used for cDNA library preparation using the NEBNext Ultra RNA Library Prep Kit for Illumina (New England Biolabs). Paired-end reads (~20 M/sample) were aligned to the hg38 human or mm10 genome using Spliced Transcripts Alignment to a Reference (STAR; v2.5) software. HTSeq v0.6.1 (45) was used to count the read numbers mapped to each gene, followed by determination of fragments per kilobase of exon model per million mapped reads (FPKM). Differential expression analysis between 2 conditions was performed using the DESeq2 R package (2_1.6.3). The resulting *P* values were adjusted using the Benjamini-Hochberg approach for controlling the false discovery rate. Genes with adjusted *P* value less than 0.05 were considered differentially expressed. Gene set enrichment analysis (GSEA) for analysis of gene expression differences was performed using the Gene Ontology and KEGG gene sets.

### Alternative splicing analysis.

Alternative splicing (AS) analysis was performed using rMATS on RNA-Seq data (46). Exon skipping, alternative 5′ splice sites, alternative 3′ splice sites, mutually exclusive exons, and retained introns were identified and compared between RMC-6272– and rapamycin-treated cells. *P* values and FDRs were calculated for differential splicing events.

### Multiomics.

Triomics analysis of lipids, metabolites, proteins, and phosphoproteins was performed as previously described (47). Pellets from 10 million cells were obtained by spinning down at 500*g* at 4°C after 24 hours of DMSO, rapamycin (10 nM), or RMC-6272 (3 nM) treatment (triplicates per condition). Two hundred microliters of 1× PBS and 1.5 mL HPLC-grade methanol were added, followed by a vigorous vortex for 1 minutes at room temperature. The samples were shaken for 1 hour at room temperature after addition of 5 mL of HPLC-grade MTBE, anhydrous 99.8% (Sigma-Aldrich, 34875-2L). Then 1.2 mL HPLC-grade water was added, vortexed for 1 minute, and spun for 10 minutes. The resulting upper (lipids) and middle (metabolites) phases were collected separately in 1.5 mL glass vials and dried out using SpeedVac Concentrator (Thermo Fisher Scientific, SC110A). A protein pellet at the bottom was used for both proteomics and phosphoproteomics. High-Select TiO_2_ Phosphopeptide Enrichment Kit (Thermo Fisher Scientific, PIA32993) was used to enrich phosphopeptides. The metabolite samples were resuspended in 20 μL liquid chromatography–mass spectrometry–grade (LC-MS–grade) water and run as previously described (48). The data were analyzed using Elements for Metabolomics (Proteome Software) with the NIST database incorporated (http://chemdata.nist.gov/mass-spc/msms-search/) followed by statistical analysis with MetaboAnalyst 5.0 (http://www.metaboanalyst.ca/). The lipid samples were resuspended in 30 μL of LC-MS–grade isopropanol/methanol (1:1), and 5 μL was injected for LC-MS/MS analysis. Lipidomic data were analyzed using LipidSearch 4.1.9 software (Thermo Fisher Scientific) and Elements for Metabolomics (Proteome Software) with NIST database incorporated. The protein samples were analyzed by positive-ion mode LC-MS/MS using a high-resolution hybrid QExactive HF Orbitrap Mass Spectrometer (Thermo Fisher Scientific) via higher-energy collisional dissociation with data-dependent analysis with 1 MS1 scan followed by 8 MS2 scans per cycle for the top 8 ions detected in the MS1 scan. MS/MS spectra were analyzed for the peptide samples with a parent ion tolerance of 18 ppm and fragment ion tolerance of 0.05 Da. Carbamidomethylation of cysteine (+57.0293 Da) was specified as a fixed modification, and oxidation of methionine (+15.9949) and phosphorylation of serine/threonine/tyrosine (+79.97) as variable modifications. Results were imported into Scaffold Q+S 4.6 software (Proteome Software Inc.) with a peptide threshold of about 85% and a protein threshold of 95%, resulting in a peptide false discovery rate of approximately 1%. Further statistical analysis was performed using PANTHER (http://www.pantherdb.org/) after removal of contaminants such as keratins, caseins, trypsin, and BSA (47). Omics data were normalized to the median of each sample and then subjected to differential analysis (DESeq2) and pathway enrichment analysis (GSEA). Lipidomic data were used to generate a lipid map using Lipid Maps (49) (https://www.lipidmaps.org).

### Immunohistochemistry.

Tumor samples were fixed for 24 hours in 10% formalin, embedded in paraffin, and sectioned (FFPE) (5 μm) for H&E and IHC staining. FFPE sections were immunostained with primary antibodies against p-S6^S240/244^ (rabbit monoclonal, CST 5364S; 1:2,000), p-4EBP1^T37/46^ (rabbit monoclonal, CST 2855S; 1:1,600), Ki-67 (rabbit monoclonal, CST 12202S; 1:400), cleaved PARP (rabbit monoclonal, CST 5625S; 1:50), and cleaved caspase-3 (rabbit monoclonal, CST 9664S; 1:1,000). Anti-rabbit secondary antibody was purchased from Vector Laboratories (MP-7401-50) and detected by ImmPACT AEC Peroxidase (HRP) Substrate Kit (SK4205). Slides were counterstained with Mayer’s hematoxylin (Agilent Technologies, S330930-2) and mounted in Fluoromount-G (SouthernBiotech, 0100-01).

### In situ cell death detection (TUNEL staining).

TUNEL staining was performed on FFPE sections. After dewaxing and hydrating, slides were permeabilized for 8 minutes at room temperature with 0.1% Triton X-100 prepared in 0.1% sodium citrate in PBS. Slides were incubated with TUNEL reaction mixture (prepared according to In Situ Cell Death Detection Kit Fluorescein, 11684795910) in a humidified atmosphere for 60 minutes at 37°C in the dark. After washing of the slides twice in PBS, DAPI (Sigma-Aldrich, D9542-10MG) was applied, and slides were mounted with Fluoromount-G.

### CUT&RUN and data analysis.

Cleavage Under Targets & Release Using Nuclease (CUT&RUN) was performed using a CUTANA ChIC/CUT&RUN Kit (EpiCypher) per the manufacturer’s protocol. One million cells were harvested for CUT&RUN. Antibodies against H3K27ac (Diagenode, C15410196) and JUN (CST 9165S) were used. Twenty million reads per sample (paired-end reads extending 150 bases) were obtained and aligned to hg38 using Bowtie 2.4.5 (50). Peaks were called using MACS3 (51). For visualization, Deeptools v3.5.0 (52) was used to convert BAM files into bigWig (bw) files. JUN ChIP-Seq data for multiple cancer cell lines were downloaded from Cistrome Data Browser (http://cistrome.org/db/#/).

### Establishing mouse LUAD cell lines.

Three pairs of mouse LUAD cell lines with *Tsc1* loss (KTP-267-1B1, KTP-267-2B8, and KTP-269-3C4) and wild-type *Tsc1* (857, 855, and 634) were generated. Briefly, *Kras^+/LSL-G12D^ Trp53^L/L^ Tsc1^fl/fl^* mice were obtained through crossing of *Kras^+/LSL-G12D^ Trp53^L/L^* with *Tsc1^fl/fl^* mice. To induce *Trp53* deletion and *Tsc1* recombination, adenovirus expressing Cre recombinase was inhaled nasally by mice at the age of 6 weeks (53). Sham adenovirus without Cre recombinase activity was used as the negative control. After 6–9 weeks of tumor development, mouse lungs were harvested, minced, and then cultured using RPMI 1640 with 10% FBS and 1% penicillin-streptomycin, supplemented with 2 mM l-glutamine. The cell lines were characterized by genotyping and Western blot (42).

### Treatment of Tsc2^+/–^ A/J mice and treatment cessation.

Sex-matched *Tsc2^+/–^* A/J mice aged 10 months were provided by TSC Alliance. Mice were treated with vehicle, rapamycin (3 mg/kg, i.p., Monday, Wednesday, Friday; LC Laboratory, R-5000), MLN0128 (0.75 mg/kg, orally, Monday–Friday; RevMed), RMC-4627 (8 mg/kg, i.p., once per week; RevMed), and RMC-6272 (8 mg/kg, i.p., once per week; ResMed). Rapamycin and MLN0128 were formulated as previously described (54). RMC-4627 and RMC-6272 were formulated in 5:5:90 (vol/wt/vol) Transcutol (Sigma-Aldrich, 537616)/Solutol HS 15 (Sigma-Aldrich, 42966)/water. Mouse body weight was measured every day. After 4 weeks of treatment, 3 mice from each group were sacrificed. The other 4 mice were kept without further treatment for 2 months for tumor regrowth. Kidneys were harvested and sectioned into 1 mm pieces along the longitudinal axis and fixed in 10% formalin. Tumor size (length and width) was measured on H&E slides under the microscope blindly. Tumor volume = maximum (tumor percent, 5)/100 × π/6 × 1.64 × (tumor length × tumor width)^1.5^ (55), where “maximum (tumor percentage, 5)” is the larger of 2 possible values, the tumor percentage in an individual cystadenoma or 5. For a solid tumor, this value is 100; for a cystic tumor, the value is 5. The total tumor volume of each kidney was calculated as the sum of all the tumor lesions.

### One-dose treatment of Tsc2^+/–^ A/J mice.

Fourteen-month-old sex-matched *Tsc2^+/–^* A/J mice (provided by TSC Alliance) were dosed once with vehicle, rapamycin (3 mg/kg, i.p.), MLN0128 (0.75 mg/kg, orally), or RMC-6272 (8 mg/kg, i.p.). Mice were sacrificed at different time points (4 hours, 1 day, 3 days, and 7 days). Mouse kidneys were harvested. Half of both left and right kidneys were fixed by 10% formalin. The other half were snap-frozen for lysates.

### Treatment of mouse lymphangioleiomyomatosis models.

TTJ cells were resuspended in 1× sterile PBS and injected into 6-week-old male C57BL/6J mice (The Jackson Laboratory, 000664) through tail vein (1 million cells per mouse). Three days after injection, mice were randomly divided into 5 groups (*n* = 10 mice per group) and treated as described above. Weights were monitored daily. After 4 weeks of treatment, 5 mice from each group were sacrificed. Mouse lungs were inflated with 4% PFA and fixed in 4% PFA for FFPE blocks. The other 5 mice were monitored for up to 2 months, without further treatment, to evaluate tumor regrowth.

### Treatment of human BLCA PDX models.

Patient-derived xenograft (PDX) model experiments were conducted by Charles River Discovery Research Services, Freiburg, Germany (for model BXF-2211) and Pharmaron, Beijing, China (for models BLC1497 and BLC1521). The *TSC1* mutation status in these 3 PDX models was validated by Western blotting or whole-exome sequencing. PDX tumors were transplanted subcutaneously into 6- to 8-week-old female immunodeficient mice. For BXF-2211, once tumor volume reached about 100–200 mm^3^, mice were randomized to receive rapamycin (3 mg/kg), MLN0128 (0.75 mg/kg), RMC-6272 (8 mg/kg), or RMC-5552 (3 mg/kg or 10 mg/kg) in a separate study, or vehicle as control. For BLC1497 and BLC1521, once tumor volume reached about 100–200 mm^3^, mice were randomized to receive RMC-5552 (3 mg/kg or 10 mg/kg) or vehicle as control. Tumors were measured twice weekly using calipers, and tumor volume was calculated using the formula 0.5 × length × width^2^. Mouse body weight was monitored twice per week.

### PDX 1-dose treatment.

The same human BLCA PDX-2211 described above was used. Mice were treated once with vehicle, rapamycin (3 mg/kg), or RMC-6272 (8 mg/kg) when tumor size reached a range of 200–600 mm^3^. Mice were sacrificed 4, 24, 72, or 168 hours after the single dose. Each tumor sample was cut into 2 pieces, with one being snap-frozen in liquid nitrogen and the other being fixed in 10% formalin for further analysis.

### Statistics.

Data were analyzed using GraphPad Prism 9.0. Results are presented as mean ± SD. All cell and Western blot experiments were repeated at least twice with separately prepared samples. The log-rank (Mantel-Cox) test was used to determine significance for survival curves. For 2-group comparisons, a 2-tailed unpaired Student’s *t* test was applied. One-way ANOVA was used for multiple comparisons in the experiments with more than 2 groups. *P* less than 0.05 was considered statistically significant. *P* values are denoted with asterisks: *P < 0.05, **P < 0.01, ***P < 0.001, and ****P < 0.0001.

### Study approval.

All animal experiments were conducted using protocols approved by the Brigham and Women’s Hospital Institutional Animal Care and Use Committee.

### Data availability.

RNA-Seq data generated in this study were uploaded into the NCBI’s Gene Expression Omnibus database (GEO GSE236742). The structure of bi-steric inhibitors used in the graphical abstract was obtained from a previous study (17). The cartoon of cell apoptosis was obtained from BioRender (https://www.biorender.com/). Values for all data points in graphs are reported in the Supporting Data Values file.

## Author contributions

HD conceived the project; developed experimental protocols; designed, performed, interpreted, and analyzed experiments; performed bioinformatics analyses; and wrote, edited, and reviewed the manuscript. YCY designed, interpreted, and analyzed experiments and edited the manuscript. HJL assisted with performance of in vivo experiments. MY and JMA performed the mass spectrometry. KKW provided the 3 pairs of mouse LUAD cell lines. EPH supervised some of the research, and reviewed and edited the manuscript. MS conceived and supervised this project and edited the manuscript. DJK conceived the project; supervised, interpreted, and analyzed experiments; reviewed all primary data; performed bioinformatics analyses; and wrote and edited the manuscript. All authors read and approved the final manuscript.

## Supplementary Material

Supplemental data

Supporting data values

## Version 1 11/01/2023

Electronic publication
