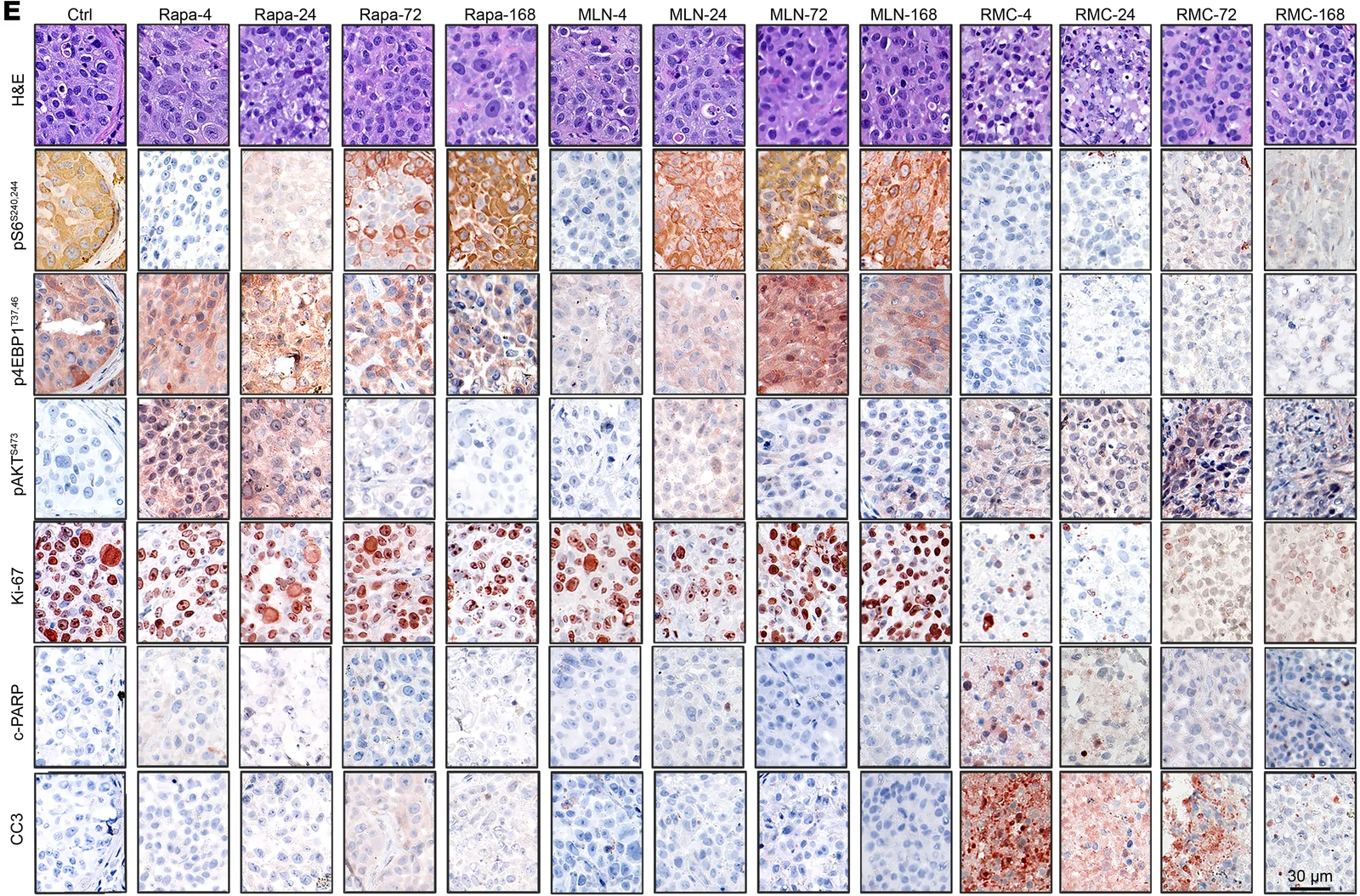# Corrigendum to Bi-steric mTORC1 inhibitors induce apoptotic cell death in tumor models with hyperactivated mTORC1

**DOI:** 10.1172/JCI213785

**Published:** 2026-11-16

**Authors:** Heng Du, Yu Chi Yang, Heng-Jia Liu, Min Yuan, John M. Asara, Kwok-Kin Wong, Elizabeth P. Henske, Mallika Singh, David J. Kwiatkowski

Original citation: *J Clin Invest*. 2023;133(21):e167861. https://doi.org/10.1172/JCI167861

Citation for this corrigendum: *J Clin Invest*. 2026;136(22):e213785. https://doi.org/10.1172/JCI213785

The authors recently became aware that in Figure 4E the c-PARP image for the 72-hour RMC treatment (RMC-72) was incorrect and was a different crop of the c-PARP image for RMC-168. The correct figure, based on the original source data, is shown below. The HTML and PDF versions of the paper have been updated online.

The authors regret the error.

## Figures and Tables

**Figure 4E:**