# Supplementary material for: Bi-steric mTORC1 inhibitors induce apoptotic cell death in tumor models with hyperactivated mTORC1
Source: J Clin Invest. 2023 Nov 1;133(21):e167861. doi: 10.1172/JCI167861 (PMC10617776; doi:10.1172/JCI167861)
Supplement: Supplemental data [file jci-133-167861-s117.pdf]

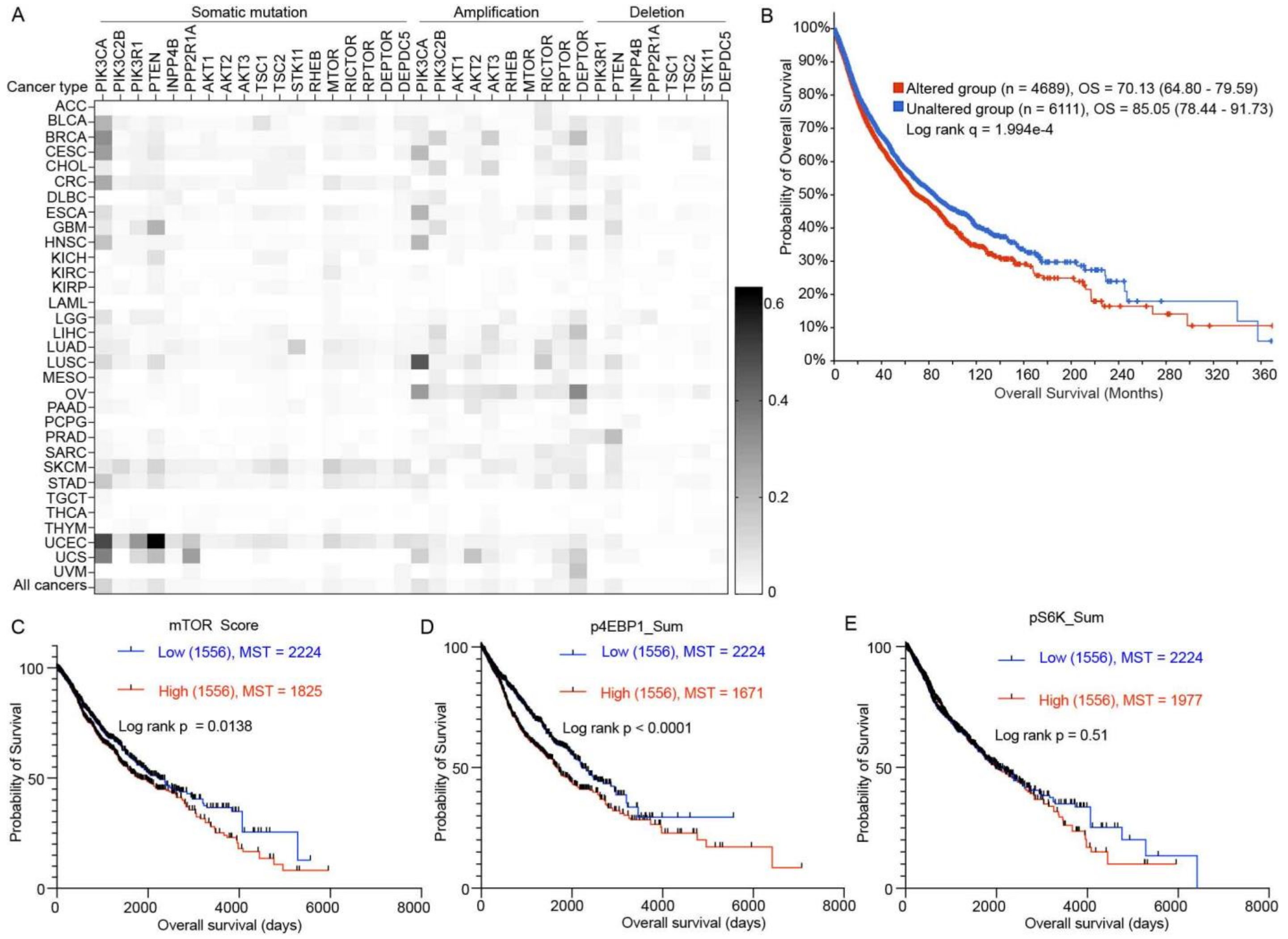

Supplementary Figure S1. mTOR is a therapeutic target in many human cancers. A. PI3K-AKT-mTOR pathway genetic alterations are common in human cancers (TCGA Pan-cancer, n = 10,800). The frequency of different types of alterations in each gene in each cancer type is shown. TCGA abbreviations are used. **B.** PI3K-AKT-mTOR pathway alterations (shown in A) are associated with a worse prognosis in TCGA tumors. **C-E.** mTOR and p4EBP1 activity, but not pS6K activity, assessed by RPPA, are associated with worse prognosis in the TCGA data set. Survival curves are shown for the top and bottom quartiles (25%) of patients according to each RPPA measure.

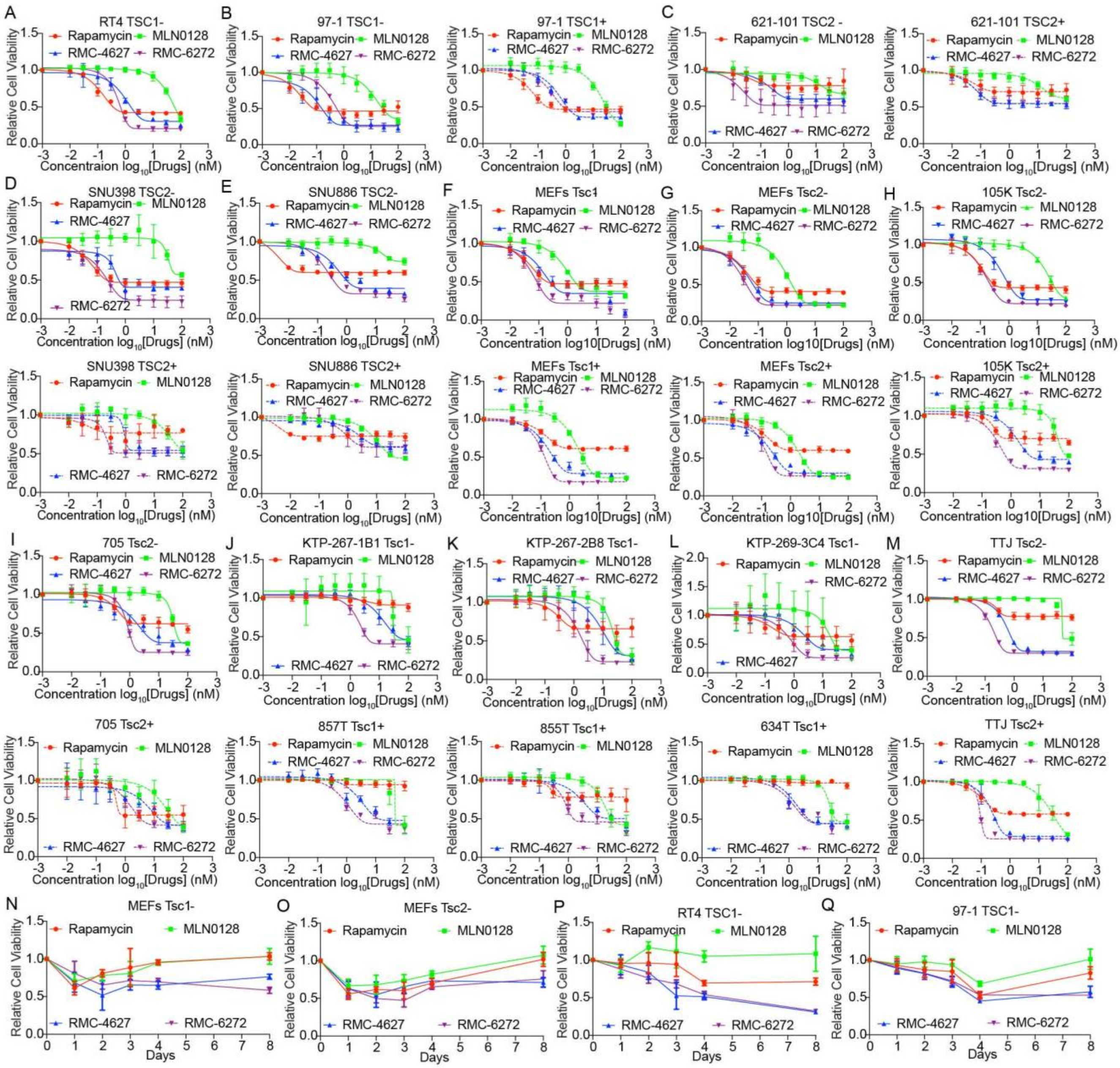

Supplementary Figure S2. Bi-steric mTOR inhibitors have a lower IC50 and stronger effect on growth than Rapamycin or MLN0128. A-N. Dose-dependent cell growth inhibition curves of TSC1 null human BLCA line (**A**, **B**), TSC2 null angiomyolipoma cell line (**C**), TSC2 null HCC cell line. (**D**, **E**), Tsc1 null and Tsc2 null murine embryo fibroblasts (MEFs) (**F**, **G**), Tsc2 null mouse RCC cell lines (**H**, **I,** **M**), mouse LUAD cell lines (**J**, **K**, **L**). Each dot and error bar on the curves represent mean ± S.D. (n = 6). **N–Q.** TSC1/TSC2 null MEFs (N, O), human BLCA cells (P, Q) were treated with Rapamycin, MLN0128, RMC-4627, and RMC-6272 for 24h (from Day -1 to Day 0). Each dot and error bar on the curves represent mean ± S.D. (n = 6).

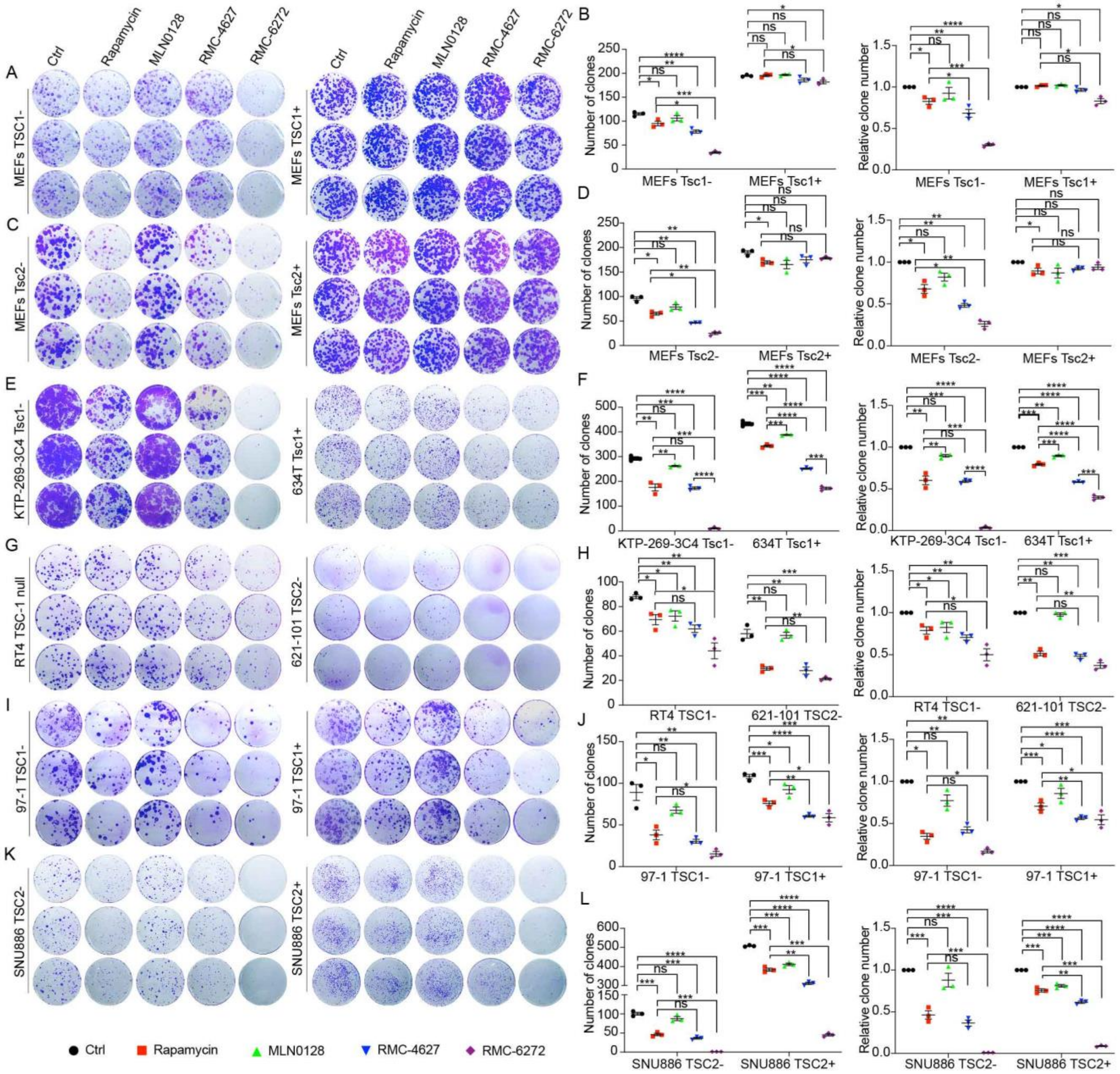

Supplementary Figure S3. Bi-steric mTOR inhibitors suppress in vitro clone formation.

A-L. TSC1/TSC2 null MEFs (**A**-**D**), mouse LUAD cells (**E**, **F**), human BLCA cells (**G**, **H, I, J**), human angiomyolipoma cells (**G**, **H**), human HCC cells (**K, L**) were plated at 1,000 cells per 10cm dish, and treated with Rapamycin, MLN0128, RMC-4627, RMC-6272 for 14 days. Both clone numbers (left) and normalized clone numbers (right) are shown in B, D, F, H, J, L. Each bar and error bar represent mean ± S.D. (n = 3). Student’s t-test was used. * $p < 0.05$, ** $p < 0.01$, *** $p < 0.001$, **** $p < 0.0001$.

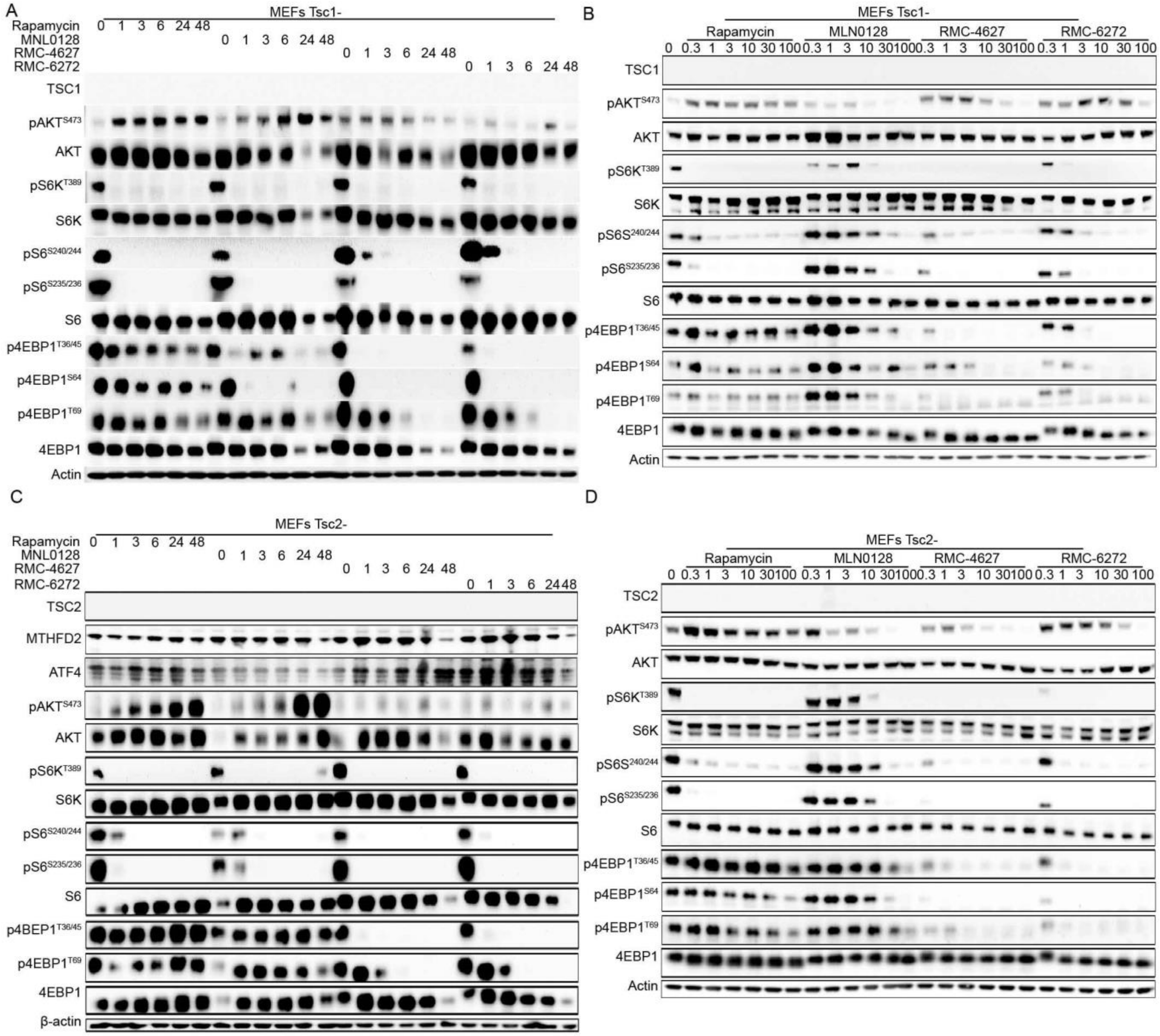

A
MEFs Tsc1-
Rapamycin 0 1 3 6 24 48
MNL0128 0 1 3 6 24 48
RMC-4627 0 1 3 6 24 48
RMC-6272 0 1 3 6 24 48
TSC1
pAKT^S473
AKT
pS6K^T389
S6K
pS6^S240/244
pS6^S235/236
S6
p4EBP1^T36/45
p4EBP1^S64
p4EBP1^T69
4EBP1
Actin
B
MEFs Tsc1-
Rapamycin
MLN0128
RMC-4627
RMC-6272
0 0.3 1 3 10 30 100 0.3 1 3 10 30 100 0.3 1 3 10 30 100 0.3 1 3 10 30 100
TSC1
pAKT^S473
AKT
pS6K^T389
S6K
pS6S^240/244
pS6^S235/236
S6
p4EBP1^T36/45
p4EBP1^S64
p4EBP1^T69
4EBP1
Actin
C
MEFs Tsc2-
Rapamycin 0 1 3 6 24 48
MNL0128 0 1 3 6 24 48
RMC-4627 0 1 3 6 24 48
RMC-6272 0 1 3 6 24 48
TSC2
MTHFD2
ATF4
pAKT^S473
AKT
pS6K^T389
S6K
pS6^S240/244
pS6^S235/236
S6
p4BEP1^T36/45
p4EBP1^T69
4EBP1
β-actin
D
MEFs Tsc2-
Rapamycin
MLN0128
RMC-4627
RMC-6272
0 0.3 1 3 10 30 100 0.3 1 3 10 30 100 0.3 1 3 10 30 100 0.3 1 3 10 30 100
TSC2
pAKT^S473
AKT
pS6K^T389
S6K
pS6S^240/244
pS6^S235/236
S6
p4EBP1^T36/45
p4EBP1^S64
p4EBP1^T69
4EBP1
Actin

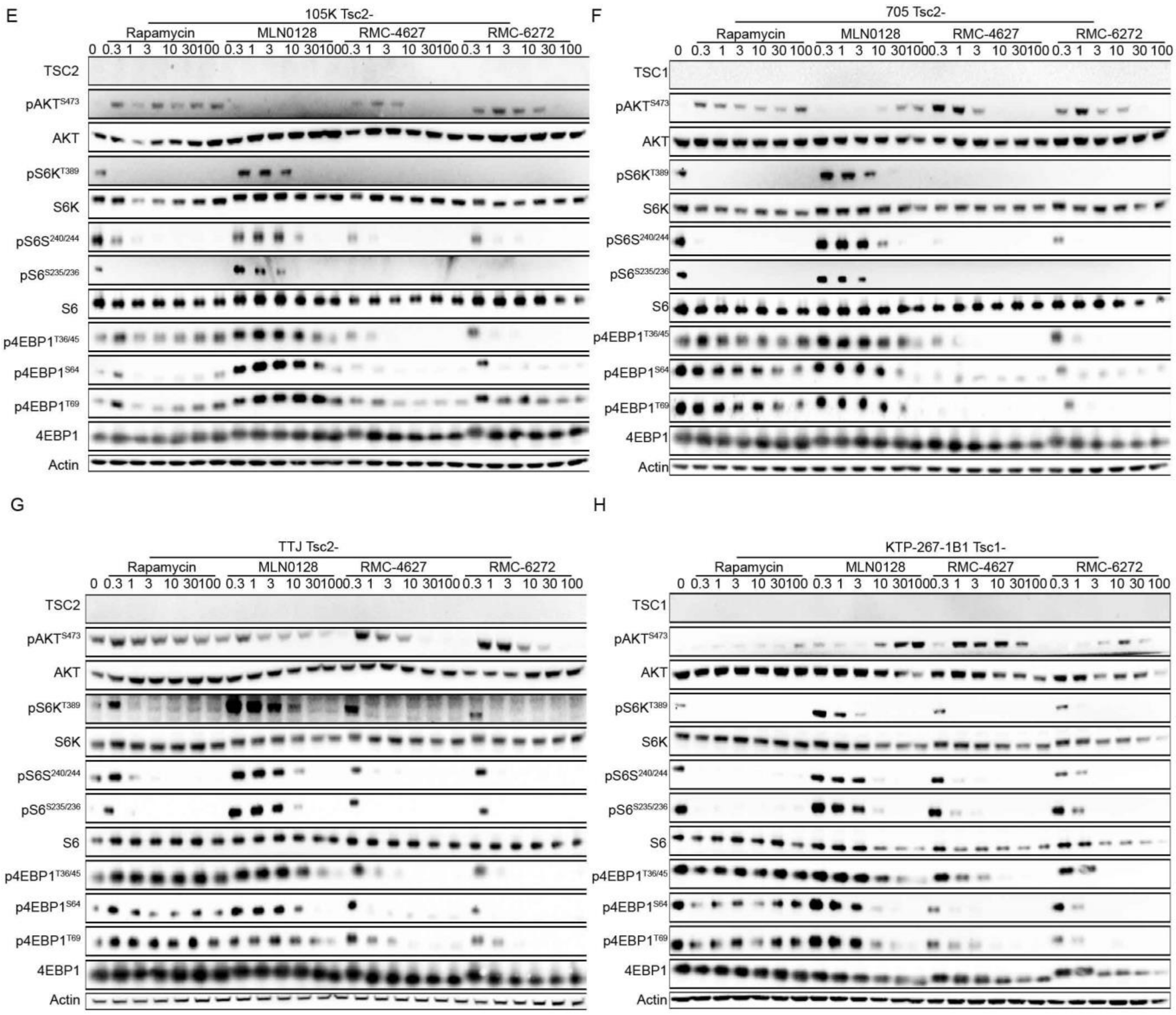
E
105K Tsc2-
Rapamycin
MLN0128
RMC-4627
RMC-6272
0 0.3 1 3 10 30100 0.3 1 3 10 30100 0.3 1 3 10 30100 0.3 1 3 10 30 100
TSC2
pAKT^{S473}
AKT
pS6K^{T389}
S6K
pS6S$^{240/244}$
pS6$^{S235/236}$
S6
p4EBP1$^{T36/45}$
p4EBP1^{S64}
p4EBP1^{T69}
4EBP1
Actin
F
705 Tsc2-
Rapamycin
MLN0128
RMC-4627
RMC-6272
0 0.3 1 3 10 30 100 0.3 1 3 10 30100 0.3 1 3 10 30100 0.3 1 3 10 30 100
TSC1
pAKT^{S473}
AKT
pS6K^{T389}
S6K
pS6S$^{240/244}$
pS6$^{S235/236}$
S6
p4EBP1$^{T36/45}$
p4EBP1^{S64}
p4EBP1^{T69}
4EBP1
Actin
G
TTJ Tsc2-
Rapamycin
MLN0128
RMC-4627
RMC-6272
0 0.3 1 3 10 30100 0.3 1 3 10 30100 0.3 1 3 10 30100 0.3 1 3 10 30 100
TSC2
pAKT^{S473}
AKT
pS6K^{T389}
S6K
pS6S$^{240/244}$
pS6$^{S235/236}$
S6
p4EBP1$^{T36/45}$
p4EBP1^{S64}
p4EBP1^{T69}
4EBP1
Actin
H
KTP-267-1B1 Tsc1-
Rapamycin
MLN0128
RMC-4627
RMC-6272
0 0.3 1 3 10 30100 0.3 1 3 10 30100 0.3 1 3 10 30100 0.3 1 3 10 30 100
TSC1
pAKT^{S473}
AKT
pS6K^{T389}
S6K
pS6S$^{240/244}$
pS6$^{S235/236}$
S6
p4EBP1$^{T36/45}$
p4EBP1^{S64}
p4EBP1^{T69}
4EBP1
Actin

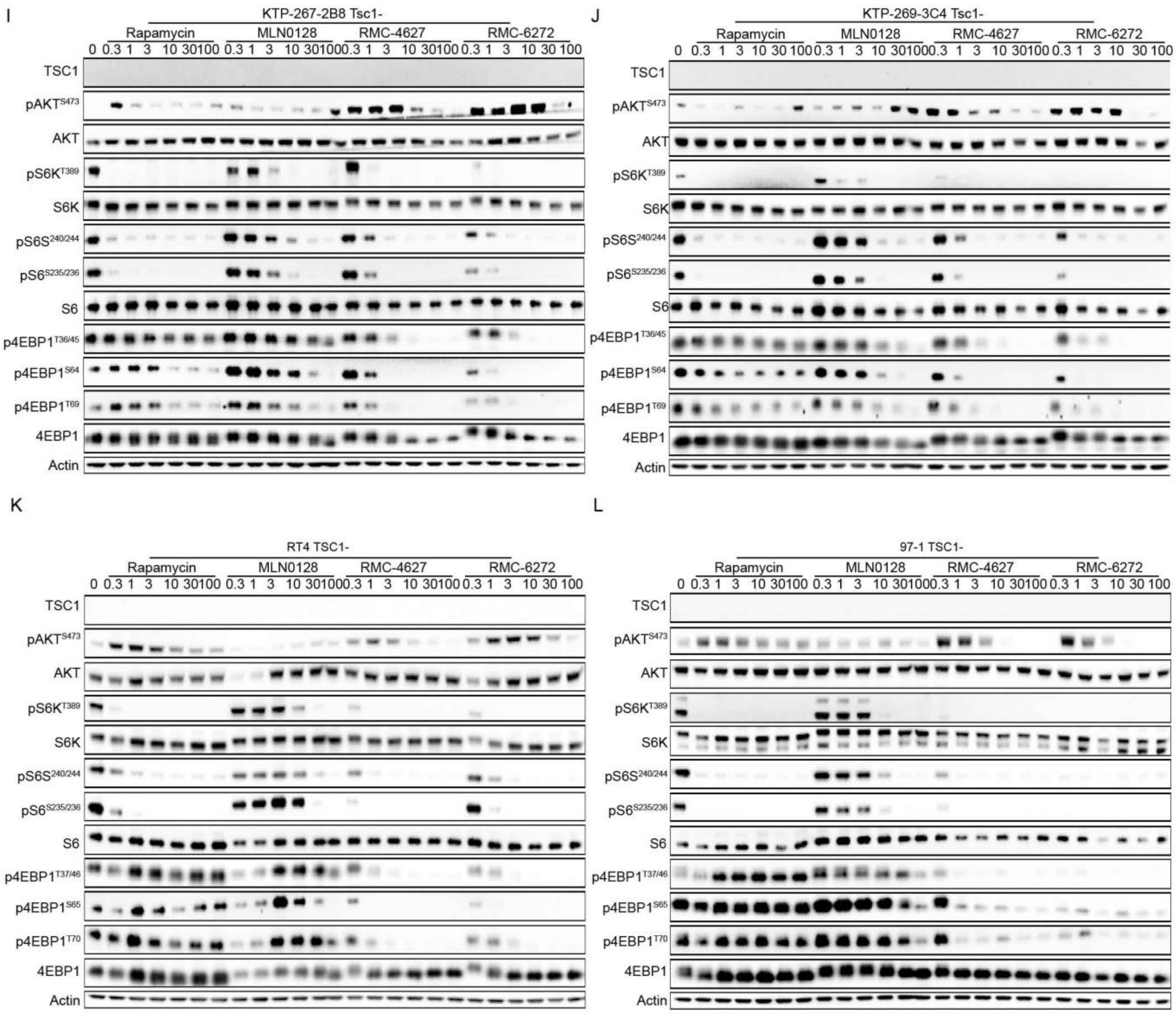

I
KTP-267-2B8 Tsc1-
Rapamycin
MLN0128
RMC-4627
RMC-6272
0 0.3 1 3 10 30100 0.3 1 3 10 30100 0.3 1 3 10 30100 0.3 1 3 10 30 100
TSC1
pAKT^{S473}
AKT
pS6K^{T389}
S6K
pS6S$^{240/244}$
pS6$^{S235/236}$
S6
p4EBP1$^{T36/45}$
p4EBP1^{S64}
p4EBP1^{T69}
4EBP1
Actin
J
KTP-269-3C4 Tsc1-
Rapamycin
MLN0128
RMC-4627
RMC-6272
0 0.3 1 3 10 30 100 0.3 1 3 10 30100 0.3 1 3 10 30100 0.3 1 3 10 30 100
TSC1
pAKT^{S473}
AKT
pS6K^{T389}
S6K
pS6S$^{240/244}$
pS6$^{S235/236}$
S6
p4EBP1$^{T36/45}$
p4EBP1^{S64}
p4EBP1^{T69}
4EBP1
Actin
K
RT4 TSC1-
Rapamycin
MLN0128
RMC-4627
RMC-6272
0 0.3 1 3 10 30100 0.3 1 3 10 30100 0.3 1 3 10 30100 0.3 1 3 10 30 100
TSC1
pAKT^{S473}
AKT
pS6K^{T389}
S6K
pS6S$^{240/244}$
pS6$^{S235/236}$
S6
p4EBP1$^{T37/46}$
p4EBP1^{S65}
p4EBP1^{T70}
4EBP1
Actin
L
97-1 TSC1-
Rapamycin
MLN0128
RMC-4627
RMC-6272
0 0.3 1 3 10 30100 0.3 1 3 10 30100 0.3 1 3 10 30100 0.3 1 3 10 30 100
TSC1
pAKT^{S473}
AKT
pS6K^{T389}
S6K
pS6S$^{240/244}$
pS6$^{S235/236}$
S6
p4EBP1$^{T37/46}$
p4EBP1^{S65}
p4EBP1^{T70}
4EBP1
Actin

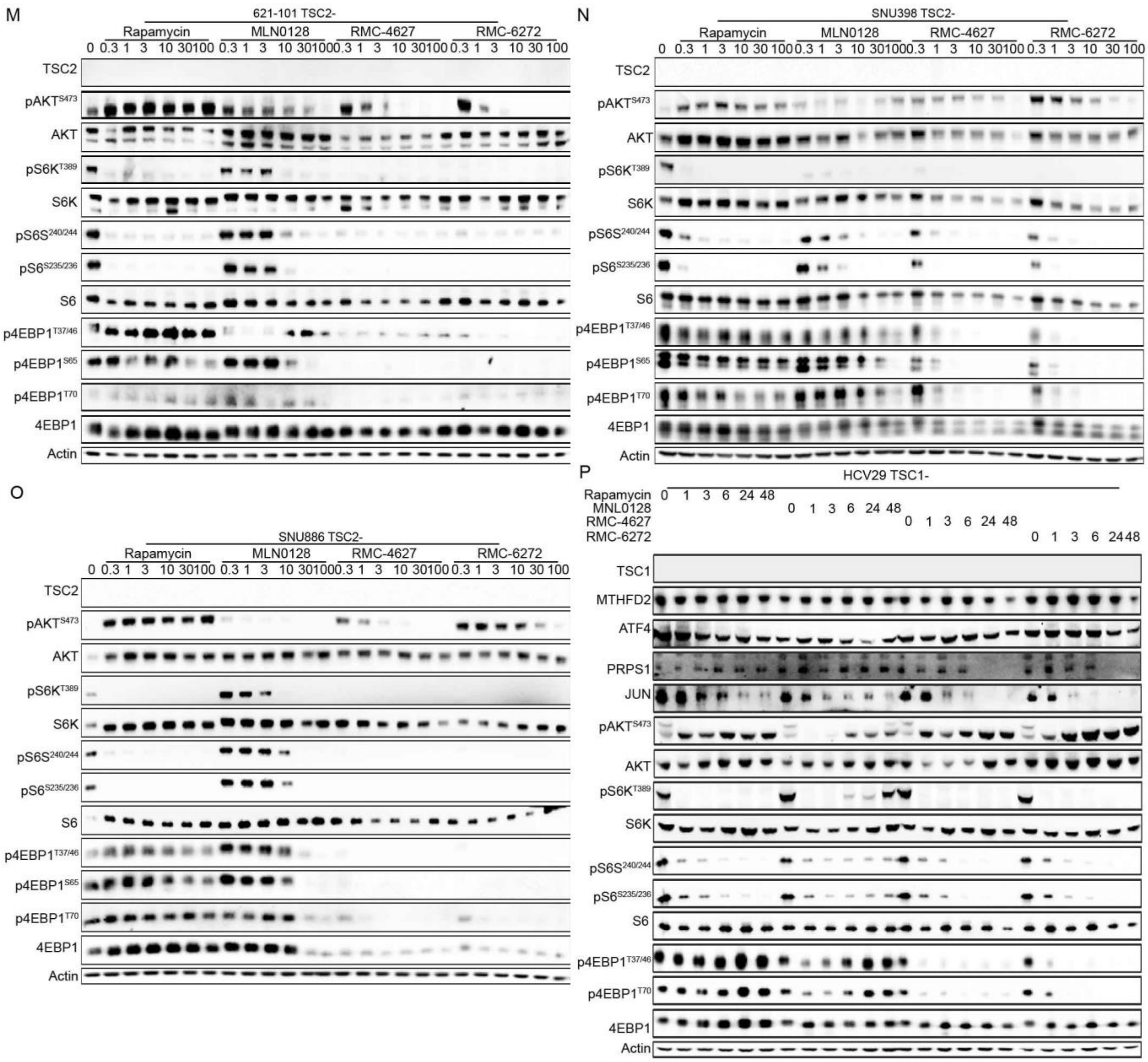

M
621-101 TSC2-
Rapamycin
MLN0128
RMC-4627
RMC-6272
0 0.3 1 3 10 30 100 0.3 1 3 10 30 100 0.3 1 3 10 30 100 0.3 1 3 10 30 100
TSC2
pAKT S473
AKT
pS6K T389
S6K
pS6S 240/244
pS6 S235/236
S6
p4EBP1 T37/46
p4EBP1 S65
p4EBP1 T70
4EBP1
Actin
N
SNU398 TSC2-
Rapamycin
MLN0128
RMC-4627
RMC-6272
0 0.3 1 3 10 30 100 0.3 1 3 10 30 100 0.3 1 3 10 30 100 0.3 1 3 10 30 100
TSC2
pAKT S473
AKT
pS6K T389
S6K
pS6S 240/244
pS6 S235/236
S6
p4EBP1 T37/46
p4EBP1 S65
p4EBP1 T70
4EBP1
Actin
O
SNU886 TSC2-
Rapamycin
MLN0128
RMC-4627
RMC-6272
0 0.3 1 3 10 30 100 0.3 1 3 10 30 100 0.3 1 3 10 30 100 0.3 1 3 10 30 100
TSC2
pAKT S473
AKT
pS6K T389
S6K
pS6S 240/244
pS6 S235/236
S6
p4EBP1 T37/46
p4EBP1 S65
p4EBP1 T70
4EBP1
Actin
P
HCV29 TSC1-
Rapamycin 0 1 3 6 24 48
MNL0128 0 1 3 6 24 48
RMC-4627 0 1 3 6 24 48
RMC-6272 0 1 3 6 24 48
TSC1
MTHFD2
ATF4
PRPS1
JUN
pAKT S473
AKT
pS6K T389
S6K
pS6S 240/244
pS6 S235/236
S6
p4EBP1 T37/46
p4EBP1 T70
4EBP1
Actin

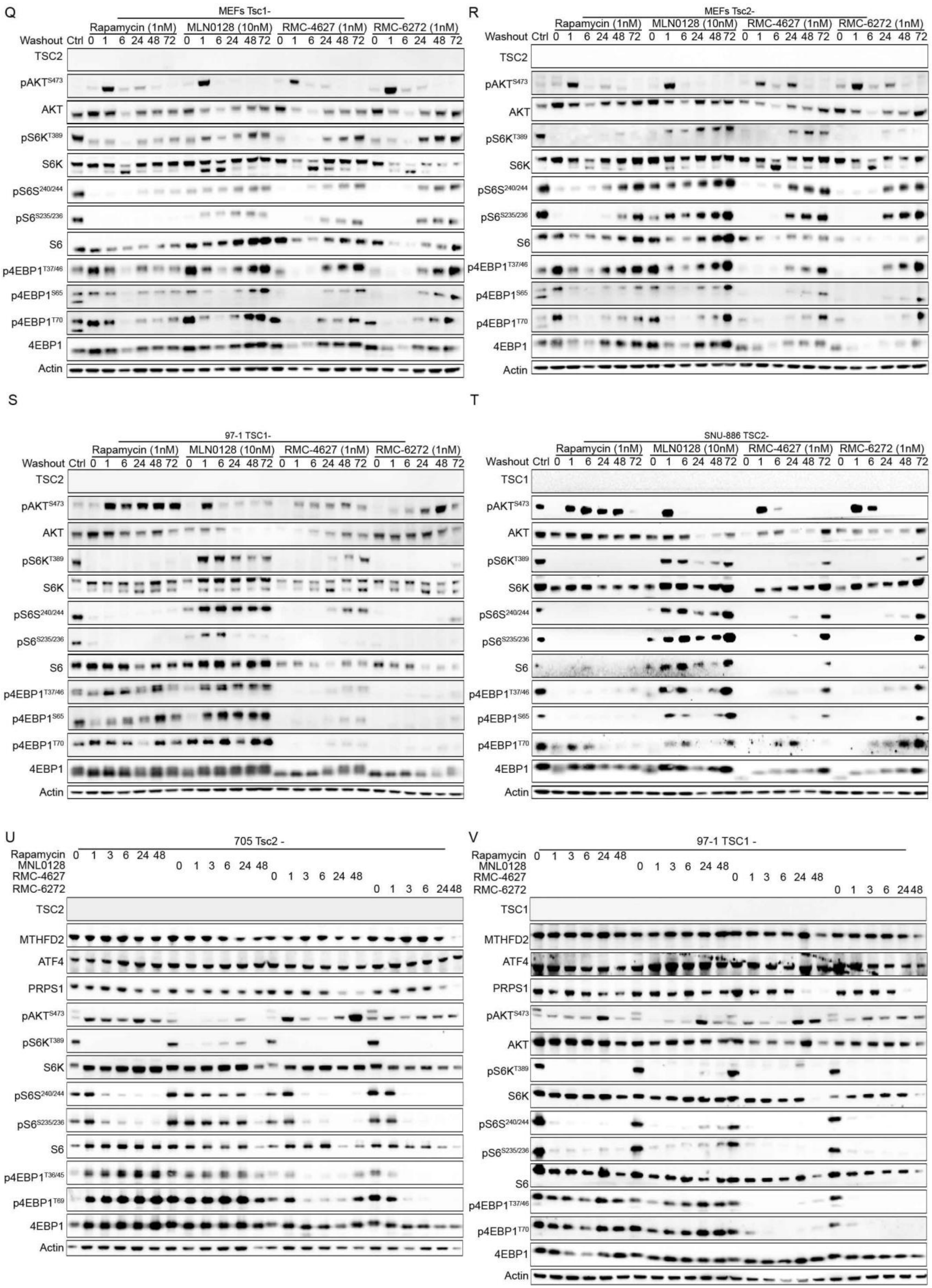
Q
MEFs Tsc1-
Rapamycin (1nM) MLN0128 (10nM) RMC-4627 (1nM) RMC-6272 (1nM)
Washout Ctrl 0 1 6 24 48 72 0 1 6 24 48 72 0 1 6 24 48 72 0 1 6 24 48 72
TSC2
pAKT^{S473}
AKT
pS6K^{T389}
S6K
pS6S$^{240/244}$
pS6$^{S235/236}$
S6
p4EBP1$^{T37/46}$
p4EBP1^{S65}
p4EBP1^{T70}
4EBP1
Actin
R
MEFs Tsc2-
Rapamycin (1nM) MLN0128 (10nM) RMC-4627 (1nM) RMC-6272 (1nM)
Washout Ctrl 0 1 6 24 48 72 0 1 6 24 48 72 0 1 6 24 48 72 0 1 6 24 48 72
TSC2
pAKT^{S473}
AKT
pS6K^{T389}
S6K
pS6S$^{240/244}$
pS6$^{S235/236}$
S6
p4EBP1$^{T37/46}$
p4EBP1^{S65}
p4EBP1^{T70}
4EBP1
Actin
S
97-1 TSC1-
Rapamycin (1nM) MLN0128 (10nM) RMC-4627 (1nM) RMC-6272 (1nM)
Washout Ctrl 0 1 6 24 48 72 0 1 6 24 48 72 0 1 6 24 48 72 0 1 6 24 48 72
TSC2
pAKT^{S473}
AKT
pS6K^{T389}
S6K
pS6S$^{240/244}$
pS6$^{S235/236}$
S6
p4EBP1$^{T37/46}$
p4EBP1^{S65}
p4EBP1^{T70}
4EBP1
Actin
T
SNU-886 TSC2-
Rapamycin (1nM) MLN0128 (10nM) RMC-4627 (1nM) RMC-6272 (1nM)
Washout Ctrl 0 1 6 24 48 72 0 1 6 24 48 72 0 1 6 24 48 72 0 1 6 24 48 72
TSC1
pAKT^{S473}
AKT
pS6K^{T389}
S6K
pS6S$^{240/244}$
pS6$^{S235/236}$
S6
p4EBP1$^{T37/46}$
p4EBP1^{S65}
p4EBP1^{T70}
4EBP1
Actin
U
705 Tsc2 -
Rapamycin 0 1 3 6 24 48
MNL0128 0 1 3 6 24 48
RMC-4627 0 1 3 6 24 48
RMC-6272 0 1 3 6 24 48
TSC2
MTHFD2
ATF4
PRPS1
pAKT^{S473}
pS6K^{T389}
S6K
pS6S$^{240/244}$
pS6$^{S235/236}$
S6
p4EBP1$^{T36/45}$
p4EBP1^{T69}
4EBP1
Actin
V
97-1 TSC1 -
Rapamycin 0 1 3 6 24 48
MNL0128 0 1 3 6 24 48
RMC-4627 0 1 3 6 24 48
RMC-6272 0 1 3 6 24 48
TSC1
MTHFD2
ATF4
PRPS1
pAKT^{S473}
AKT
pS6K^{T389}
S6K
pS6S$^{240/244}$
pS6$^{S235/236}$
S6
p4EBP1$^{T37/46}$
p4EBP1^{T70}
4EBP1
Actin

Supplementary Figure S4. Assessment of mTORC1 signaling effects of bi-steric mTOR inhibitors in multiple tumor cell lines. A-O. Immunoblot analysis of TSC1/TSC2 null MEFs (**A-D**), mouse RCC cells (**E-G**), mouse LUAD cells (**H-J**), human BLCA cells (**K**, **L, P**), human angiomyolipoma cells (**M**), human HCC cells (**N**, **O**) were treated with Rapamycin, MLN0128, RMC-4627, RMC-6272. **A, C**, **P** all treatments were with 20nM for variable time periods, as indicated. **B**, **D**-**O**, treatments were at variable doses (nM) for 4h. **Q-T.** TSC1/TSC2 null MEFs (**Q, R**), human BLCA cells (**S**), human HCC cells (**T**) were treated with Rapamycin, MLN0128, RMC-4627 and RMC-6272 for 24h, followed by washout for 3 days. 705 and HCV29 cells were treated with Rapamycin, MLN0128, RMC-4627, RMC-6272 for different timepoints **(U, V)**.

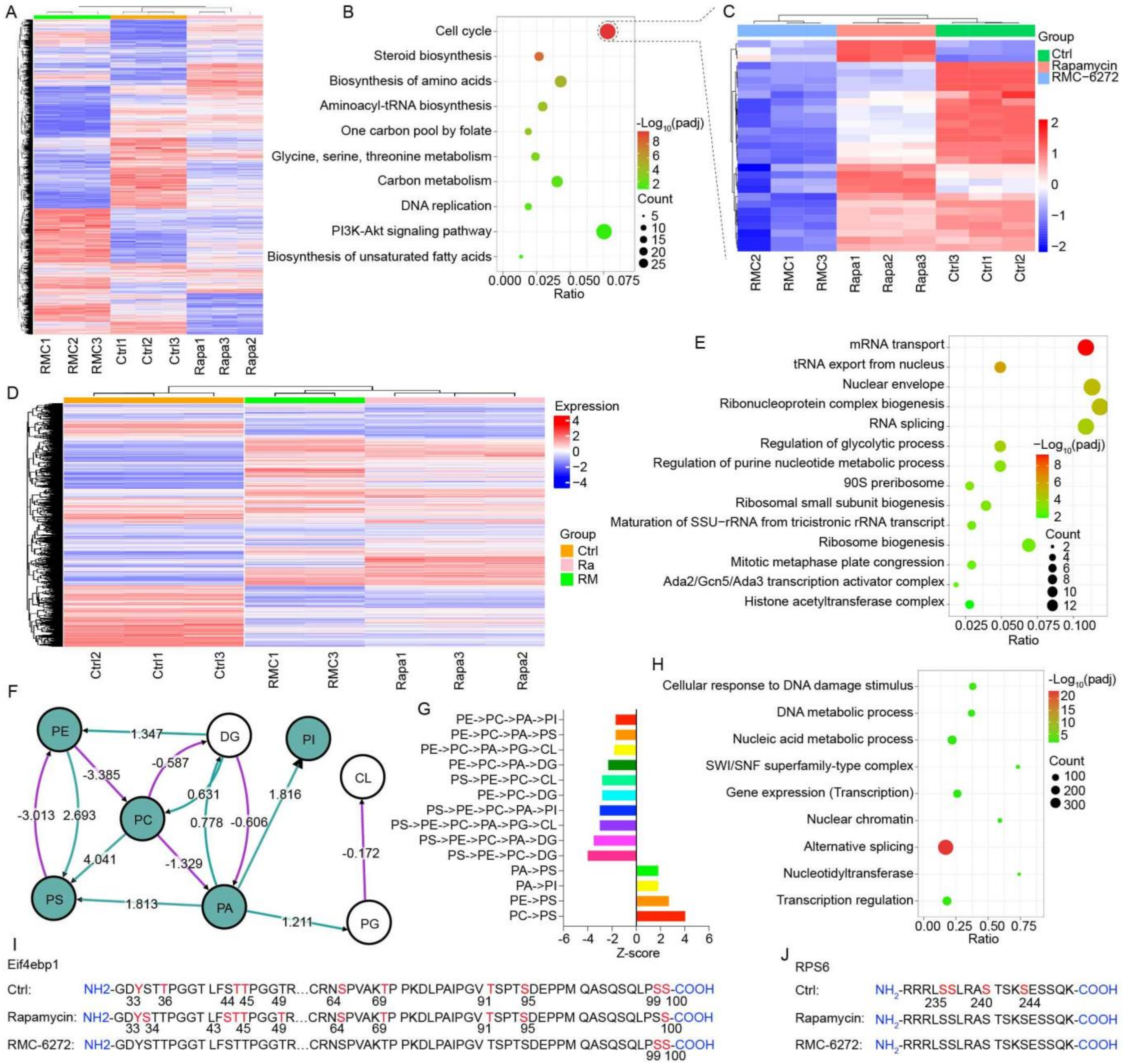

Supplementary Figure S5. Multi-omic analysis of effects of RMC-6272 vs. rapamycin. A. Heat map of RNA-seq data for the HCV29 cells treated with DMSO, Rapamycin (10 nM), or RMC-6272 (1 nM) for 24h. **B.** Pathways that are significantly enriched for genes whose expression was decreased comparing RMC-6272 to Rapamycin treatment. **C.** Heat map of those genes involved in cell cycle regulation that were decreased by RMC-6272. **D.** Heat map of mRNA levels in 705 cells treated with RMC-6272, rapamycin, or control. **E.** Global proteomic analysis showed that proteins involved in mRNA transport, RNP biogenesis, and RNA splicing pathways were selectively downregulated by RMC-6272 compared to Rapamycin in 705 Tsc2-null cells. **F.** Lipid maps showing changes in levels of different lipids comparing RM-6272- with rapamycin- treated 705 RCC cells. **G.** Lipid reaction chains/flows for RMC-6272-treated 705 cells vs. rapamycin-treated which were significantly suppressed (negative Z-score) or activated (positive Z-score), $p < 0.05$ equivalent to $|Z\text{-score}| > 1.645$. **H.** Pathway analysis of the genes showing changes in alternative splicing in response to RMC-6272 vs. rapamycin showed enrichment in an Alternative Splicing gene set. **I and J.** Phospho-proteomic analysis identified modified phospho-sites in Eif4ebp1 (**I**) and Rps6 (**J**). Phosphorylated amino acids identified under each condition are in red font.

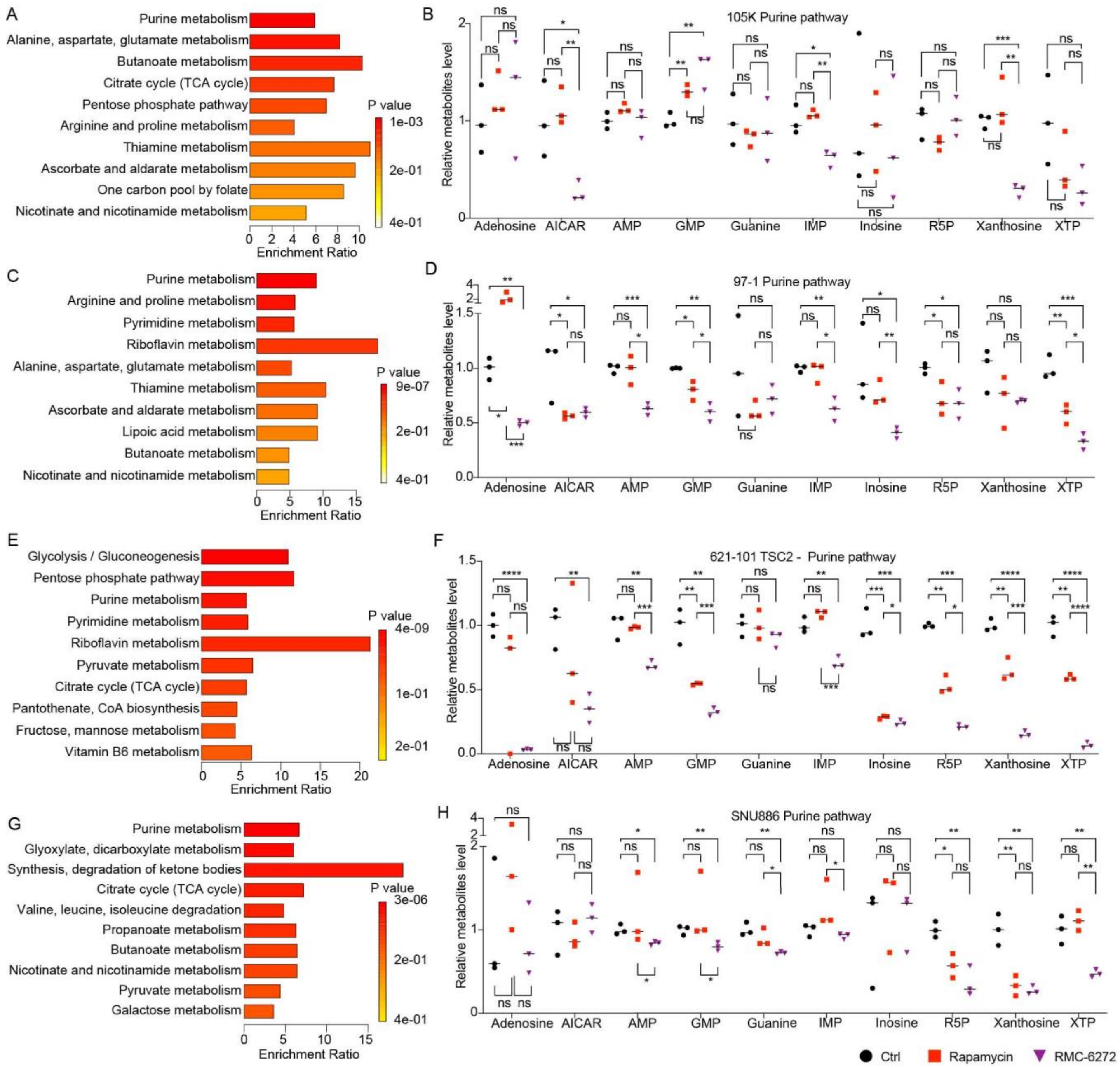

Supplementary Figure S6. Decrease in purine metabolites in multiple TSC1/TSC2 null cell lines with RMC-6272 treatment. A-H. MSEA shows that purine metabolism is one of the top enriched pathways for metabolite decrease, comparing RMC-6272 treatment with rapamycin treatment in mouse RCC cells (105K Tsc2-null) (**A**, **B**), human BLCA cells (97-1 TSC1-null) (**C**, **D**), human angiomyolipoma cells (621-101 TSC2-null) (**E**, **F**) and human HCC cells (SNU-886 TSC2-null) (**G**, **H**). Enrichment ratio: the number of metabolites that were significantly decreased within each pathway. Dots represent individual values; median line is shown (n = 3). Student's t-test was used. * $p < 0.05$, ** $p < 0.01$, *** $p < 0.001$, **** $p < 0.0001$.

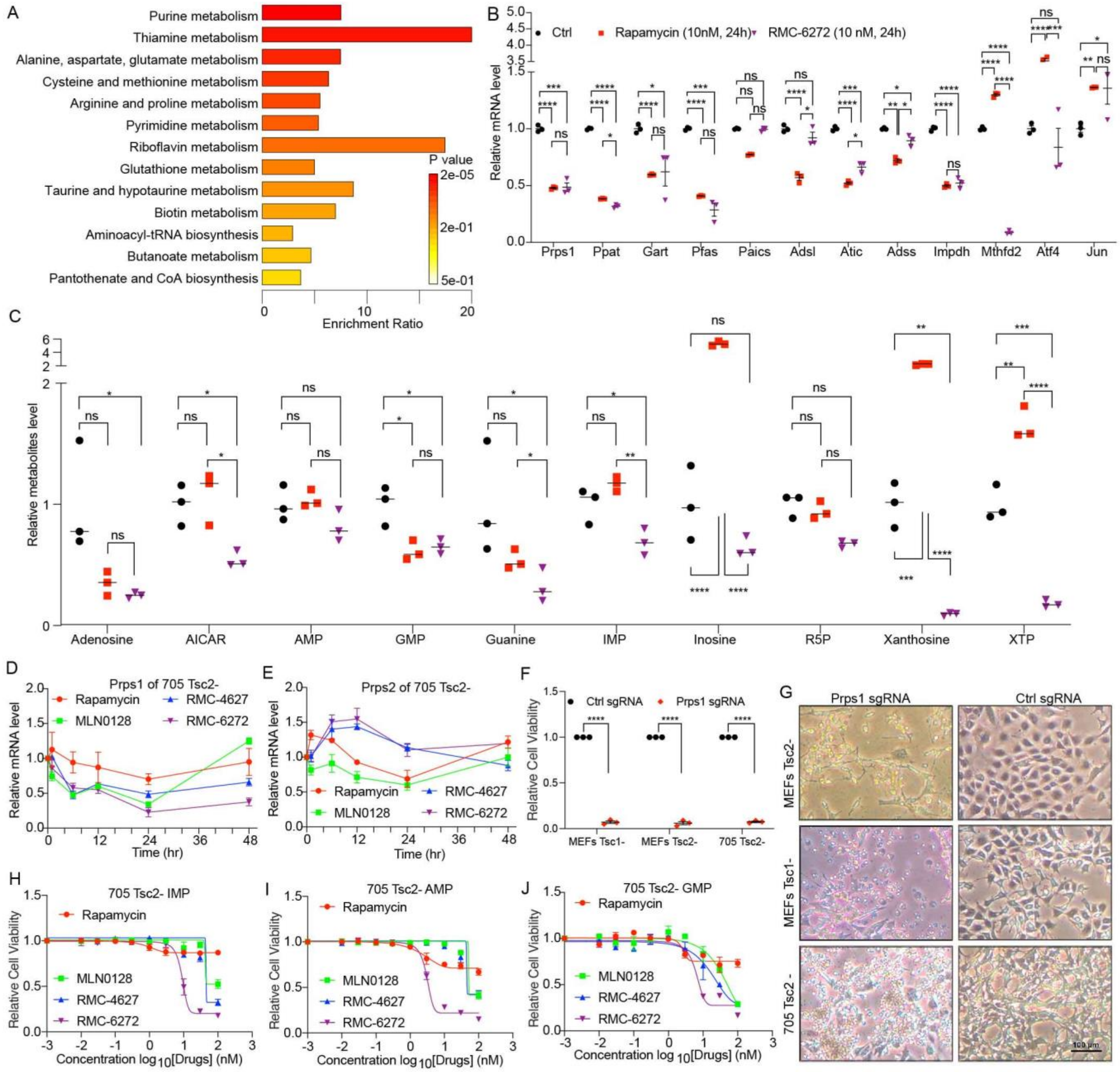

Supplementary Figure S7. De novo purine synthesis is downregulated by RMC-6272 and regulated by PRPS1 downstream of mTORC1-JUN. A. MSEA showing purine metabolism was the pathway most enriched for decreased metabolites by RMC-6272 compared to Rapamycin treatment in Tsc2-null 705 RCC cells. **B.** mRNA levels (RNA-seq data) of de novo purine synthesis enzymes and related TFs in treated 705 Tsc2-null cells. Each bar/dot and error bar represent mean ± S.D. (n = 3). Student's t-test was used. **C.** Bar graph showing purine metabolites following RMC-6272 and/or rapamycin treatment of 705 Tsc2-null cells. Dots are individual values; a median line is shown. Each bar/dot represents median (n = 3). Student's t-test was used. **D and E.** Q-RT-PCR assessment of the changes of Prps1 (**D**) and Prps2 (**E**) mRNA levels in 705 Tsc2-null cells treated with Rapamycin (10 nM), MLN0128 (10 nM), RMC-4627 (3 nM) or RMC-6272 (3 nM) for different times. **F and G.** Relative cell viability and phase contrast photos showing that cell apoptosis was induced by completely knocking out Prps1 in Tsc1 null or Tsc2 null MEFs and Tsc2 null mouse RCC cells. **H-J** IC50 of mouse RCC cells (705 Tsc2-null) supplied with IMP (**F**), AMP (**G**), GMP (**H**) and treated with Rapamycin, MLN0128, RMC-4627, and RMC-6272. Each bar/dot and error bar represent mean ± S.D. (n = 6).

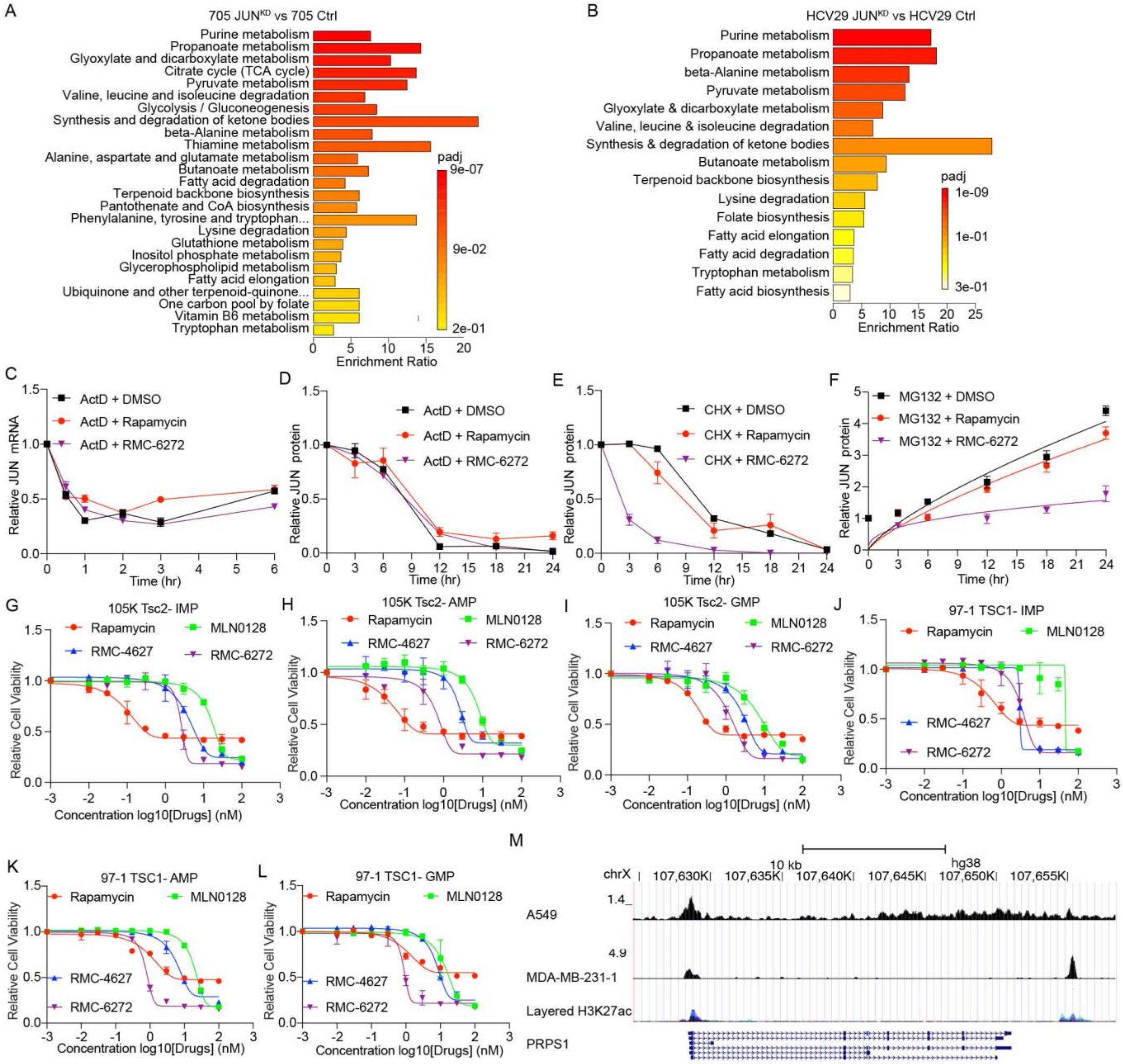

Supplementary Figure S8. De novo purine synthesis is regulated by JUN, downstream of mTORC1 activity. A, B. MSEA analysis of 705 Tsc2-null and HCV29 TSC1-null cells with JUN KD. **C – F.** JUN mRNA (by Q-RT-PCR, **CD**) and protein levels (assessed by WB, **EF**) in TSC1-/- HCV29 cells treated with 1 ug/ml Actinomycin D (**CD**), 10 uM CHX (**E**) or 2 uM MG132 (**F**) and 10 nM Rapamycin or 3 nM RMC-6272 for different time points. Each bar/dot and error bar represent mean ± S.D. (n = 3). **G – L.** IC50 curves of 105K and 97-1 cell lines supplemented with IMP (**G, I**), AMP (**H, K**), GMP (**I, L**) and treated with Rapamycin, MLN0128, RMC-4627, and RMC-6272. **M.** PRPS1 is a downstream target of JUN as shown by JUN ChIP-seq in A549 (lung cancer) and MDA-MD-231 (breast cancer) cells (CISTROME).

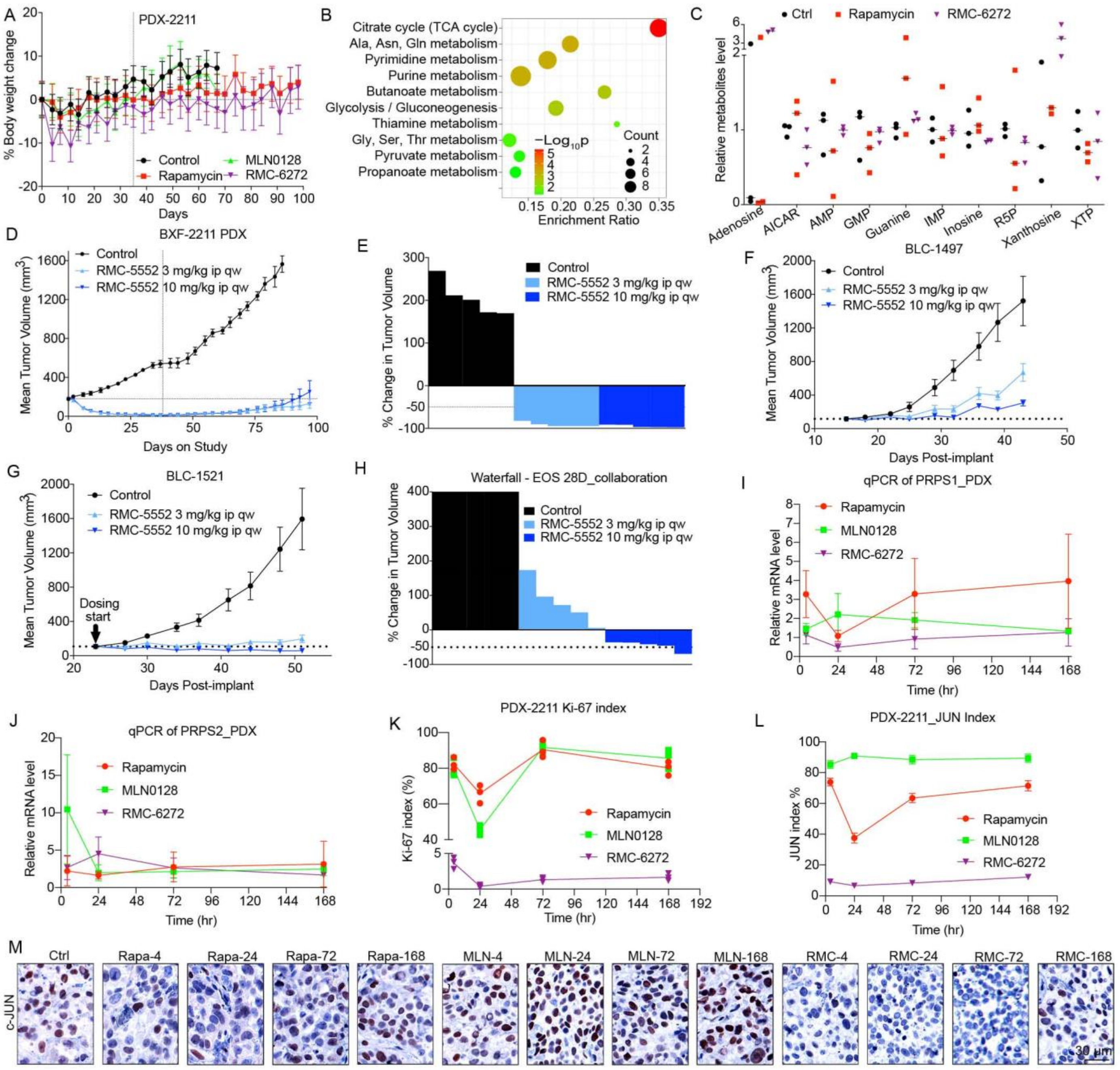

Supplementary Figure S9. Effects of bi-steric compounds in additional PDX models. A. Bodyweight change of human BLCA PDX-2211. **B, C.** Purine metabolism did not show an apparent decrease after dosing once (Rapamycin or RMC-6272) and harvested after 4h. Dots are individual values (n=3); a median line is shown. All metabolites showed no significant difference. **D-H.** RMC-5552, another bi-steric inhibitor, showed variable tumor suppression in three different human TSC1-null BLCA PDX models, PDX-2211 (**D, E**), PDX-1497 (**F**), and PDX-1521 (**G, H**). **I, J.** Bi-steric inhibitor caused a dramatic decrease of PRPS1 compared to Rapamycin or MLN0128, while PRPS2 goes up with all three drugs in one-dose treated PDX-2211, by Q-RT-PCR. Each bar/dot represents mean ± S.D. (n = 3). **K, L.** Quantification of Ki-67 and JUN IHC staining in one-dose treated PDX-2211. Ki-67 or JUN positive cells were counted and divided by the total number of cells counted. **M.** IHC staining of JUN on tumors harvested from one-dose treated PDX-2211 (same as **B**, **C**).

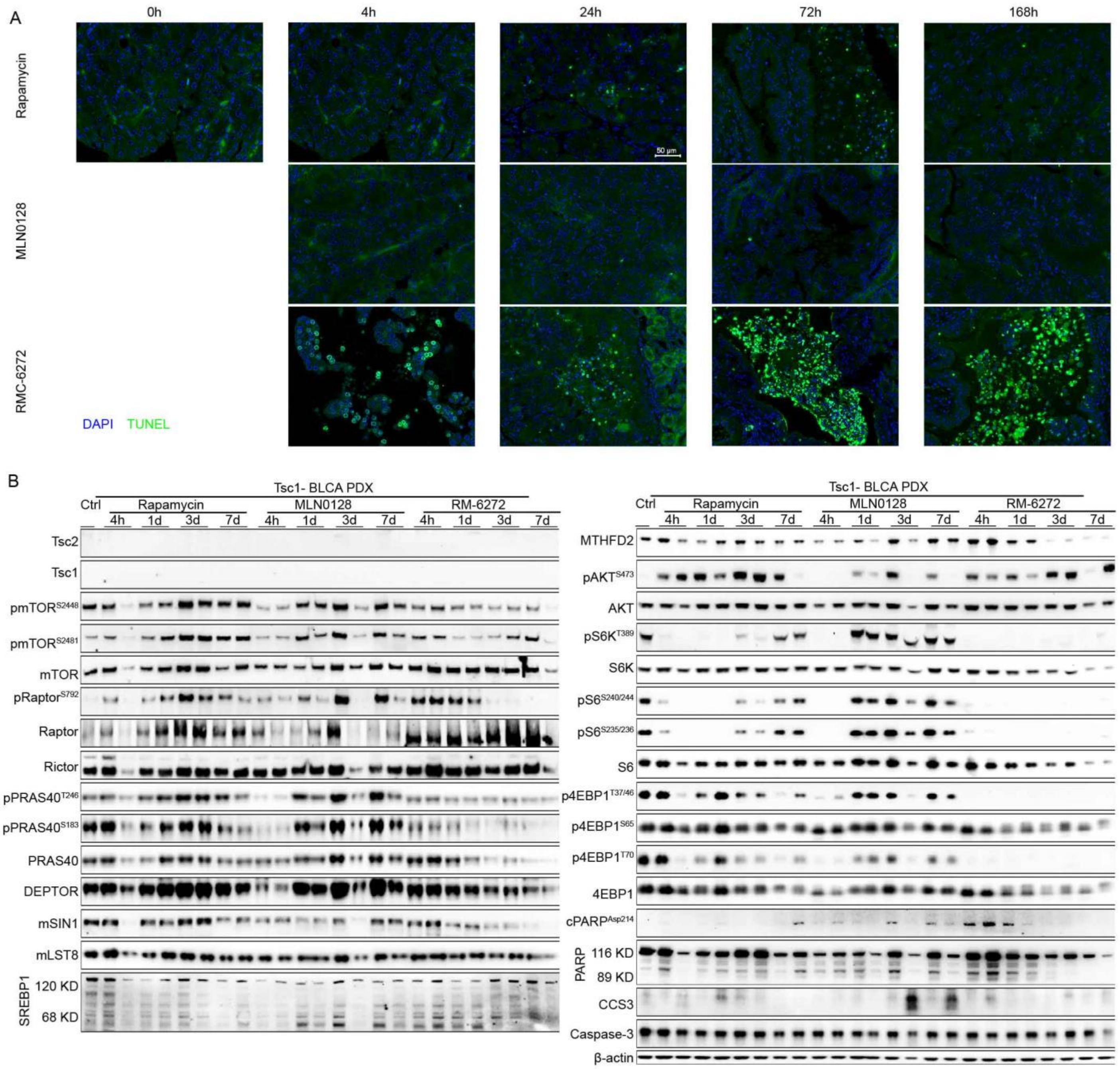

Supplementary Figure 10. Bi-steric inhibitors induced dramatic cancer cell apoptosis. A. TUNEL staining of kidneys from mice treated with single dose of Rapamycin (3 mg/kg), MLN0128 (0.75 mg/kg), and RMC-6272 (8 mg/kg) and harvested at different timepoints as indicated. TUNEL: green; DAPI: blue. Scale bar = 50 um. **B.** WB of BLCA PDX dosed once followed by washout for 4, 24, 72, and 168.

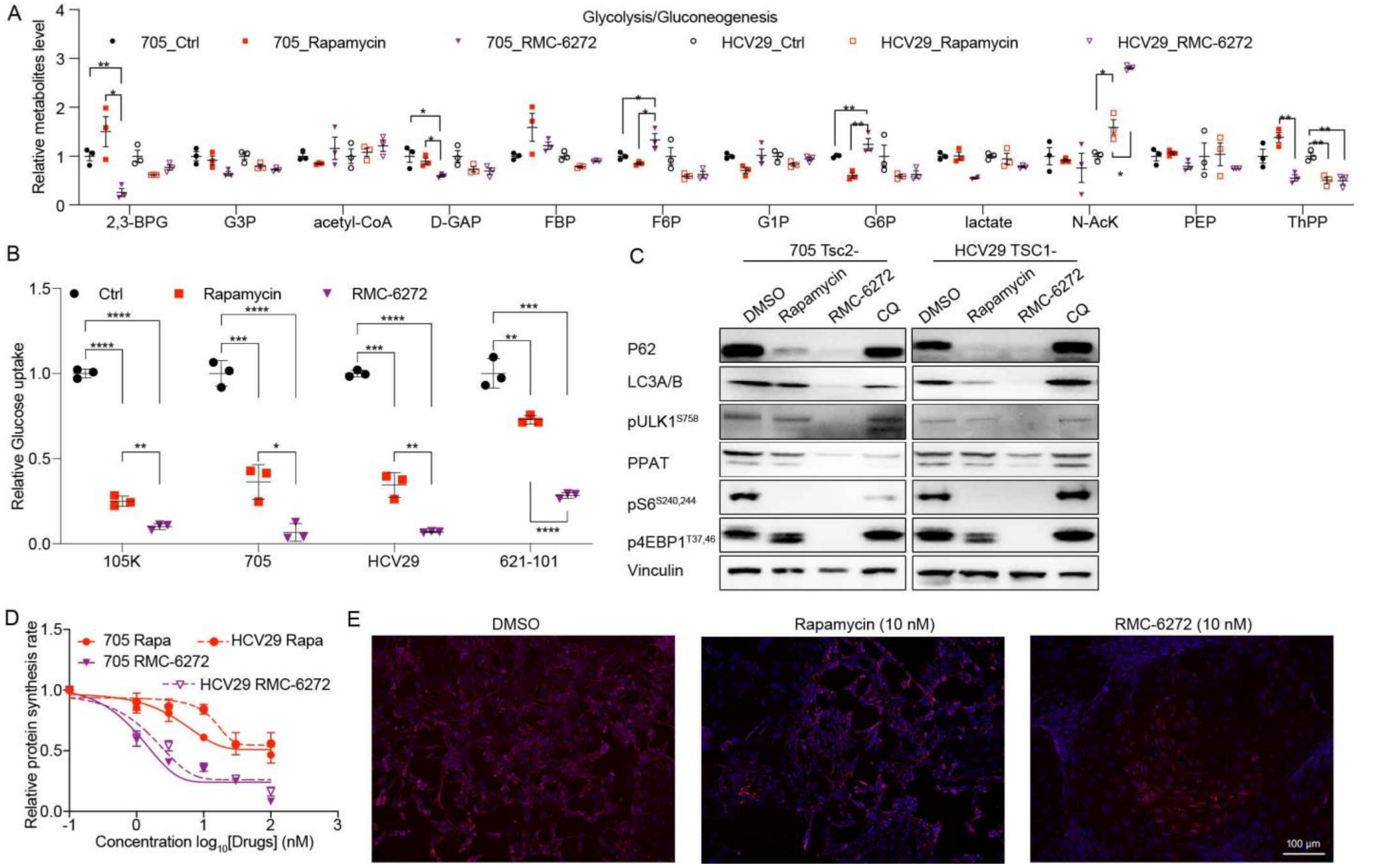

Supplementary Figure 11. Bi-steric inhibitors have a broad effect on glucose metabolism, autophagy, and protein synthesis. A. Metabolites involved in glycolysis show no significant difference between bi-steric and Rapamycin treatment. **B.** Glucose consumption was measured by the rate of glucose update. Both Rapamycin and RMC-6272 caused a significant reduction of glucose uptake in comparison to DMSO treated cells, but RMC-6272 had a stronger effect, nearly 90% reduction. **C.** RMC-6272 leads to more pronounced autophagy than rapamycin, as judged by p62 and LC3 expression. **D and E.** Protein synthesis rate was differentially suppressed by bi-steric inhibitor in comparison to Rapamycin (mean ± S.D., n = 4).

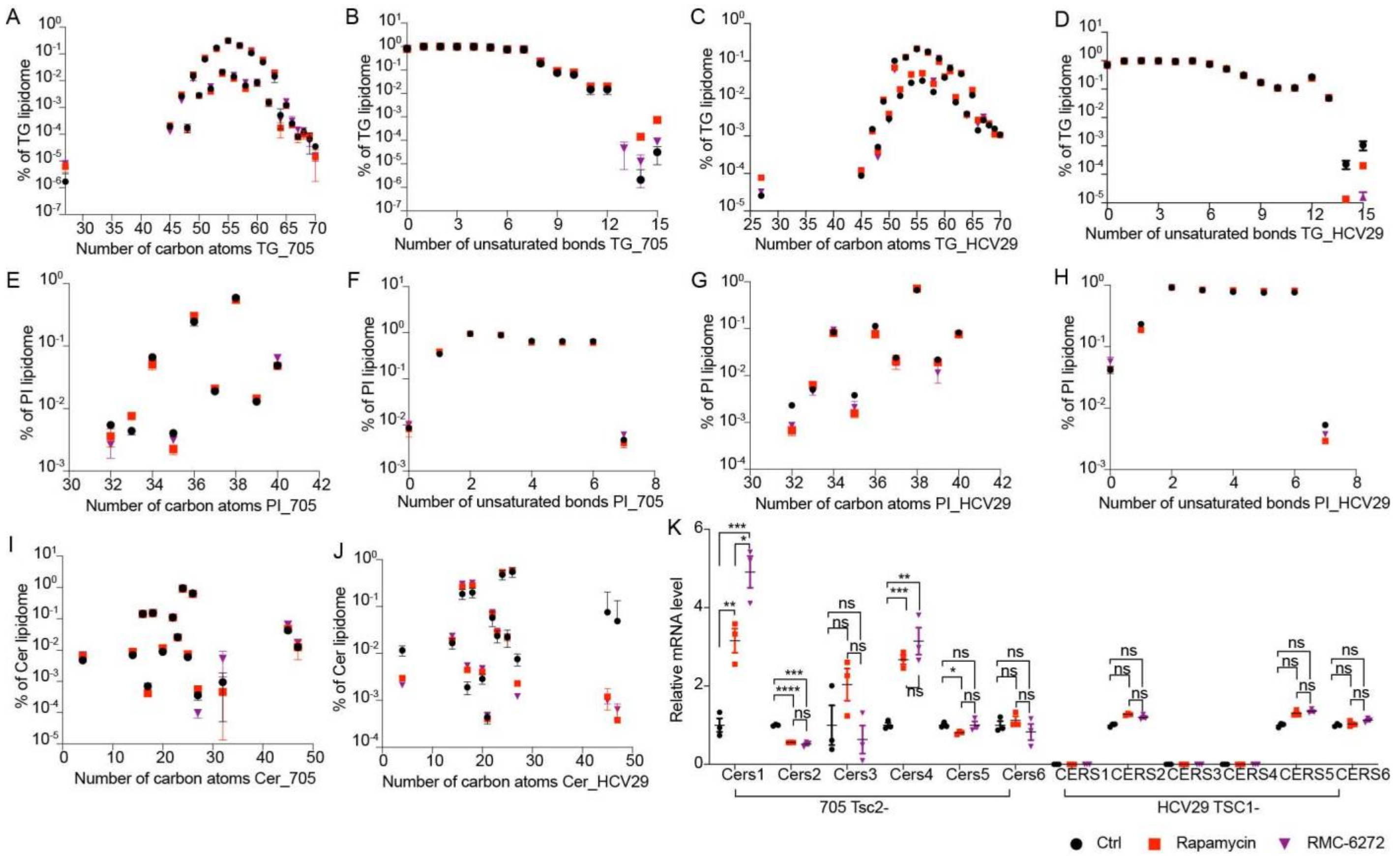

Supplementary Figure 12. Bi-steric inhibitors and rapamycin have similar effects on most lipid classes, but differential effects on ceramide synthases. A – J. The length of carbon chain and the number of unsaturated bonds of TG, PI, and Cer were determined using lipidomic data from 705 and HCV29 cell lines. Each dot represents the percent of a subclass of lipid with a specific length of carbon chain or a specific number of unsaturated bonds (mean ± S.D., n = 3). **K.** mRNA levels of Ceramide Synthases, CERS1 – CERS6, in 705 and HCV29 cell lines in response to treatment with Bi-steric inhibitors and rapamycin (mean ± S.D., n = 3).

Supplementary Table 1. IC50 values for all cell lines treated with different conditions.

	HCV29 TSC1-	HCV29 TSC1+	HCV29 TSC1- IMP	HCV29 TSC1- AMP	HCV29 TSC1- GMP	
Rapamycin	0.08	NA	NA	NA	NA	
MLN0128	31.62	35.48	10.00	10.00	5.75	
RMC-4627	0.05	0.32	0.63	0.32	0.76	
RMC-6272	0.02	0.08	0.19	0.10	0.14	
	97-1 TSC1-	97-1 TSC1+	97-1 TSC1- IMP	97-1 TSC1- AMP	97-1 TSC1- GMP	
Rapamycin	0.08	0.32	2.00	4.68	NA	
MLN0128	39.81	46.77	69.18	22.39	22.91	
RMC-4627	0.13	1.41	4.47	8.91	12.59	
RMC-6272	0.50	0.68	5.25	0.83	1.00	
	RT4 TSC1-	RT4 TSC1+	621-101 TSC2-	621-101 TSC2+	TTJ Tsc2-	TTJ Tsc2+
Rapamycin	0.32	NA	NA	NA	NA	NA
MLN0128	63.10	NA	NA	NA	63.10	50.12
RMC-4627	1.58	NA	NA	NA	0.71	0.32
RMC-6272	0.63	NA	0.14	NA	0.21	0.10
	SNU398 TSC2-	SNU398 TSC2+	SNU886 TSC2-	SNU886 TSC2+		
Rapamycin	0.20	NA	NA	NA		
MLN0128	NA	NA	NA	44.67		
RMC-4627	0.44	NA	1.32	NA		
RMC-6272	0.20	1.00	0.30	NA		
	105K Tsc2-	105K Tsc2+	105K Tsc2- IMP	105K Tsc2- AMP	105K Tsc2- GMP	
Rapamycin	0.32	NA	0.34	0.18	0.32	
MLN0128	41.69	79.43	19.95	14.13	12.59	
RMC-4627	1.00	3.98	5.62	4.47	4.37	
RMC-6272	0.20	0.56	3.16	1.00	1.91	
	705 Tsc2-	705 TSC2+	705 Tsc2- IMP	705 Tsc2- AMP	705 Tsc2- GMP	
Rapamycin	NA	NA	NA	NA	NA	
MLN0128	50.12	50.12	NA	56.23	63.10	
RMC-4627	3.98	15.85	56.23	56.23	31.62	
RMC-6272	1.00	3.55	12.59	6.31	7.08	
	KTP-267-1B1 Tsc1-	857T Tsc1+	KTP-267-2B8 Tsc1-	855T Tsc1-	KTP-269-3C4 Tsc1-	634T Tsc1-
Rapamycin	NA	NA	NA	NA	NA	NA
MLN0128	44.67	70.79	28.18	31.62	26.92	60.26
RMC-4627	52.48	17.78	21.88	NA	4.68	6.61
RMC-6272	4.17	3.16	1.78	2.00	1.00	5.62

Supplementary Table S2. mRNA of the genes in cell cycle pathway (related to Figure 2B).

SYMBOL	HCV29 _Ctrl_1	HCV29 _Ctrl_2	HCV29 _Ctrl_3	HCV29_Ra pamycin_1	HCV29_Ra pamycin_2	HCV29_Ra pamycin_3	HCV29_RMC-6272_1	HCV29_RMC-6272_2	HCV29_RMC-6272_3
TUBB4B	374.62	381.24	385.12	264.32	264.17	262.54	105.13	108.05	102.33
ANLN	85.28	84.75	86.87	60.13	63.06	59.11	21.87	22.00	23.24
TPX2	191.09	196.00	193.43	110.49	115.42	107.76	45.06	49.06	47.20
KANK2	18.27	17.65	19.04	32.27	32.35	32.68	13.82	14.13	13.90
CCL2	16.71	17.11	16.31	35.30	34.51	33.11	6.78	6.69	7.36
CDKN2C	23.58	24.53	24.69	27.21	28.30	30.86	7.55	7.44	6.34
AURKA	86.45	92.22	91.00	52.25	52.20	51.00	21.78	20.05	18.84
UBE2C	155.05	148.48	146.05	121.67	122.81	123.35	53.41	56.02	55.26
E2F1	26.25	26.33	26.40	29.68	27.99	31.11	8.39	8.64	7.35
CENPF	57.41	57.74	59.03	39.38	40.48	36.36	20.58	20.71	21.72
CDC20	159.10	151.09	153.10	88.12	82.72	90.69	42.30	40.22	40.21
GTSE1	30.56	31.83	31.86	21.44	21.40	21.78	8.37	7.48	8.15
FAM83D	56.45	55.61	58.78	35.72	35.17	35.87	12.92	13.09	11.14
PCNA	68.52	71.89	71.62	71.18	71.91	71.08	29.22	28.13	31.52
BUB1	37.33	39.48	38.13	21.89	22.56	21.98	10.31	11.04	10.32
TFDP1	55.36	57.47	56.25	44.89	45.13	43.52	23.30	23.83	22.96
KIF14	28.20	28.45	28.17	20.84	20.27	19.76	10.36	10.18	10.03
CDK1	31.75	34.99	34.05	30.24	29.89	29.35	11.52	9.72	12.44
SLFN11	13.86	14.01	14.34	16.07	16.63	15.58	6.91	6.82	6.39
CCNB1	107.52	109.60	108.73	51.72	54.48	50.52	24.95	26.40	26.55
DLGAP5	36.08	36.10	33.66	22.63	20.74	20.97	8.97	8.49	7.87
BUB1B	19.37	20.26	21.52	13.40	13.79	13.40	4.82	4.95	5.23
MAPRE1	142.86	136.99	142.32	124.37	127.80	125.73	79.65	81.99	84.85
CLSPN	5.57	5.17	5.27	5.17	5.96	5.27	1.25	1.03	1.28
DTL	12.03	13.33	13.72	13.08	13.60	13.22	5.46	5.53	5.89
ESPL1	12.59	12.28	12.27	8.99	9.26	8.77	4.35	4.22	4.55
RCC2	47.53	46.83	48.07	39.15	37.79	37.97	22.34	23.73	23.91
CDKN1A	66.11	66.44	66.83	94.21	94.28	98.23	65.42	66.19	62.28
TUBG1	46.46	45.77	43.41	37.30	37.77	39.18	17.99	19.45	21.03
PLK2	149.64	146.31	148.99	173.66	172.05	170.85	134.47	134.10	136.50
CDC6	6.95	7.42	7.21	6.56	6.10	6.39	2.04	2.39	2.14
ATP2B4	27.04	26.87	25.96	25.66	26.28	25.00	17.68	17.72	17.92
PLK1	41.17	38.31	38.25	22.00	23.14	22.36	13.25	13.20	13.20
SPDL1	19.73	20.39	20.73	15.93	16.43	16.20	8.27	7.52	8.80
CCND3	11.53	10.26	10.93	11.89	11.91	13.65	6.01	6.01	6.28
DDX3X	22.61	23.71	22.87	20.80	20.32	18.65	13.53	13.54	13.65
CDK2AP2	29.30	25.95	29.38	32.51	31.25	35.53	16.56	16.28	16.98
MAD2L1	12.56	13.85	13.34	11.66	12.33	12.21	6.54	5.32	5.82
CENPE	9.79	10.57	9.83	6.43	7.09	6.39	3.08	3.16	3.49
CDK2	9.25	8.83	9.65	8.64	7.76	8.90	2.76	2.80	3.39

TUBA4A	34.35	33.64	32.57	25.42	27.72	27.87	15.83	16.58	15.35
PSMD1	50.31	51.22	50.76	43.62	44.47	43.33	29.00	30.97	31.22
NEK2	33.20	31.38	31.32	25.52	24.65	22.25	13.35	11.91	12.59
CDKN2D	10.16	8.98	9.45	18.75	19.95	20.39	7.38	7.44	5.74
CDCA5	30.72	30.40	30.35	26.38	25.52	27.16	16.67	15.95	15.46
H2AFY	10.42	10.09	10.53	8.66	8.52	8.46	5.88	5.73	5.56
PSMC3	122.47	120.64	117.90	110.90	111.27	111.90	83.13	80.49	79.74
NDC80	22.59	22.03	23.54	14.51	16.05	13.79	7.31	6.78	7.19
HMMR	18.28	17.50	17.91	17.60	17.94	16.57	9.90	8.68	9.16
PRKACA	12.41	11.92	11.79	12.37	11.29	11.95	6.00	6.04	7.00
PSMA4	22.10	20.27	19.26	17.39	17.48	16.18	11.20	10.34	11.02
PSMB2	35.80	36.59	37.23	29.14	28.12	27.12	18.55	19.42	19.04
E2F8	2.77	3.22	3.03	3.30	3.31	3.42	0.90	0.79	0.84
MDM2	18.33	19.23	18.89	20.83	21.59	20.23	13.65	15.17	15.41
TUBA1A	11.51	11.30	13.35	14.70	15.11	14.96	7.34	8.04	8.54
CDC45	7.52	8.14	7.73	9.22	7.83	7.53	2.91	2.99	3.25
HSP90AA1	125.37	128.09	126.55	100.68	104.11	99.65	78.56	81.38	82.79
RFWD3	18.58	18.60	18.01	14.48	14.19	14.32	8.54	9.32	9.58
ID2	8.44	8.14	7.56	9.63	9.65	8.49	2.57	3.69	2.61
PSME3	40.96	43.98	42.00	35.30	34.54	35.10	25.08	26.01	26.08
CNOT6	9.58	9.73	10.14	9.57	9.80	9.71	5.84	5.25	6.05
ACTR1A	33.08	34.04	32.42	32.17	31.08	32.94	22.67	23.65	22.10
FBXO5	8.60	8.63	8.40	8.16	8.32	8.14	3.96	4.21	4.24
BRCA1	8.15	7.99	8.03	6.38	6.28	6.11	3.78	3.61	4.01
CKAP5	43.80	44.99	44.84	31.48	30.90	29.28	22.38	23.37	24.08
DONSON	12.05	11.42	13.24	9.81	11.56	11.01	5.93	5.32	5.62
EZH2	7.05	7.75	7.79	7.99	8.53	7.25	4.24	3.38	4.04
CDC25C	5.25	4.76	5.37	5.85	6.27	5.70	2.47	2.60	2.33
PSMB5	89.15	81.58	81.41	85.18	91.26	95.91	67.45	62.12	59.05
TRIP13	15.55	16.21	15.66	13.58	14.22	13.87	8.42	8.05	8.49
PSMD14	26.26	24.13	24.88	19.17	18.89	18.86	13.16	13.40	14.62
RBL1	10.24	8.92	10.16	7.85	8.37	8.22	5.09	4.52	4.76
PSMB3	117.82	100.35	109.15	95.54	97.05	102.09	67.16	71.70	69.79
NDE1	11.75	11.71	10.79	10.01	9.73	10.56	6.52	6.08	5.72
CDC25A	3.26	3.51	3.28	2.76	3.20	2.77	0.87	0.97	0.85
ERCC2	13.25	13.34	14.60	15.71	15.44	16.31	10.63	11.25	11.38
RAB11A	20.18	21.79	20.04	22.08	22.08	21.92	16.71	16.12	15.75
CEP78	2.81	3.22	2.86	2.49	2.42	2.75	1.35	1.46	1.43
CCP110	3.72	3.52	3.08	4.33	4.28	4.14	2.22	2.62	2.55
RDX	35.93	36.40	35.88	36.78	35.51	34.71	28.50	29.00	29.24
TMOD3	8.40	9.10	8.43	9.59	10.12	9.93	6.90	7.39	7.17
TUBB	454.73	442.81	456.48	348.93	353.85	344.27	303.18	309.53	303.47
RPA2	17.67	18.73	17.84	19.46	20.62	19.03	11.99	12.12	11.40

WEE1	6.00	6.15	6.21	8.60	8.70	9.09	6.36	5.95	6.25
CDC27	34.52	34.92	35.87	30.63	31.54	28.96	22.57	22.75	24.73
CCNE1	2.52	2.51	3.02	5.91	5.47	5.16	1.87	2.57	1.77
PSMD10	12.02	12.95	13.53	13.52	13.63	13.81	8.00	8.58	7.16
TAOK2	11.50	11.81	12.53	12.62	12.54	13.89	9.71	9.75	9.42
CDC7	6.91	5.81	6.28	5.24	4.51	3.79	1.61	1.70	2.12
CNOT9	24.81	25.22	24.99	19.01	18.46	18.30	13.59	13.48	14.25
DBF4B	7.08	7.57	7.64	4.01	4.42	4.25	2.43	2.56	2.24
DCTN2	33.38	35.92	36.13	38.07	36.68	39.00	30.40	29.12	29.68
PCNT	7.05	6.42	6.61	6.56	6.39	6.24	4.68	4.61	4.16
TNKS1BP1	36.02	35.65	35.61	28.78	29.48	30.55	24.64	22.68	23.05
PSMC4	41.84	40.13	38.57	36.40	37.18	36.69	26.59	25.49	28.24
CDK5RAP2	7.70	8.29	8.07	6.59	7.32	6.81	4.72	5.00	4.74
FHL1	9.11	8.85	8.03	12.19	11.50	10.58	7.45	7.93	8.07
SKP1	8.40	8.31	8.03	9.09	9.46	8.78	7.03	7.02	6.74
KNTC1	4.66	4.48	4.31	3.57	3.70	3.53	2.25	2.40	2.37
PSME2	24.70	23.64	22.49	21.82	22.47	23.55	17.14	16.01	16.45
DDB1	34.18	33.46	33.89	37.46	36.89	36.47	32.28	31.56	32.44
BID	10.46	9.50	9.86	11.95	14.18	12.95	8.84	8.70	9.16
AURKB	34.24	32.73	33.90	21.29	22.03	22.73	15.86	14.83	13.31
FZR1	14.85	13.75	14.80	11.96	12.47	13.59	9.61	8.83	8.67
ANAPC11	9.61	8.78	9.19	12.52	12.20	12.71	9.33	8.20	7.65
TTK	11.82	13.09	12.14	9.36	9.49	8.89	6.25	6.55	6.02
PSMB6	62.74	56.08	55.99	51.91	55.90	58.50	41.79	42.93	37.70
PSMD11	28.90	27.61	28.04	19.38	18.43	18.22	14.62	15.37	15.11
CDC25B	26.61	26.92	26.94	15.76	15.47	16.19	11.90	12.21	11.22
DBF4	10.25	9.65	10.38	4.92	5.68	4.81	2.90	2.89	3.26
TP53	27.46	27.91	27.78	36.10	34.68	36.37	30.04	30.64	28.48
YWHAE	160.98	157.45	152.32	158.07	158.69	149.42	133.47	136.95	138.69
KLHL22	3.75	3.54	4.22	5.53	5.07	4.81	3.38	2.92	3.06
NEDD1	10.79	10.37	12.00	10.46	10.44	8.79	6.53	6.41	7.19
BUB3	30.51	30.13	30.00	36.04	35.02	36.55	30.98	31.00	31.24
TICRR	3.03	2.99	2.85	2.74	2.70	2.76	1.63	1.74	1.75
CETN2	15.21	14.13	11.60	19.74	18.28	16.00	12.55	10.32	11.70
ATAD5	3.03	3.01	2.95	2.30	2.50	2.23	1.43	1.34	1.13
PLK4	3.92	4.05	4.05	3.14	2.81	2.82	1.11	1.42	1.76
TRIAP1	18.85	17.25	17.57	20.35	20.87	19.64	14.62	12.60	12.59
YWHAG	66.25	70.93	63.92	52.14	52.95	51.25	42.82	44.67	45.53
HAUS1	12.47	13.77	12.99	11.68	12.54	12.26	7.75	7.62	8.65
RBX1	12.94	12.63	10.76	11.05	11.19	12.38	8.36	8.22	7.15
RPS27L	11.49	9.54	8.72	11.23	12.69	11.90	9.77	9.27	9.78
AKT1	7.11	7.15	7.14	7.23	6.71	7.51	5.82	5.64	4.93
TGFB1	25.45	25.59	24.59	35.93	33.44	36.24	26.97	28.23	30.29

PSMA3	30.83	34.18	31.68	28.27	28.00	26.46	21.73	22.41	22.62
STXBP4	0.74	0.72	0.71	0.63	0.64	0.50	0.26	0.35	0.28
RAD51C	5.01	5.65	5.22	3.98	3.76	3.30	2.61	2.08	2.35
ANAPC5	10.60	10.91	10.57	11.30	10.80	10.70	9.24	9.57	9.05
GEN1	2.71	2.74	2.68	1.91	2.05	2.04	1.52	1.24	1.32
PSMD3	48.79	51.46	47.85	43.70	44.49	47.52	39.36	40.33	38.47
ODF2	8.76	9.40	8.76	8.93	8.88	7.74	6.08	6.69	6.82
PIDD1	7.72	7.56	8.03	7.04	6.91	7.79	5.86	5.07	5.42
CLASP1	8.38	8.25	8.21	7.49	8.01	7.66	6.13	6.18	6.75
SOX4	3.29	3.45	3.45	8.60	8.65	8.68	7.15	6.54	6.84
RAD21	46.59	46.48	46.80	44.82	45.38	43.91	39.46	38.96	41.28
RIPK1	10.78	11.53	11.06	11.19	11.85	11.60	8.71	9.26	9.36
PSMD13	34.38	33.67	33.80	33.20	30.71	33.86	28.85	28.05	26.79
PSME1	53.97	52.81	51.23	61.20	62.85	65.66	53.00	52.04	55.86
BAX	49.33	50.68	49.39	52.75	54.90	58.01	49.51	45.69	45.89
BLM	2.12	2.56	2.33	2.10	1.85	2.25	1.20	1.34	1.35
PSMB1	56.11	63.11	54.12	65.63	69.85	63.51	56.64	53.81	59.45
CEP250	10.97	11.08	11.22	9.23	9.12	9.00	8.01	7.86	8.12
E2F4	25.78	25.53	25.65	19.33	19.83	20.55	16.13	16.39	17.02
CENPJ	2.51	2.46	2.44	2.04	2.68	1.97	1.61	1.19	1.23
CUL3	12.08	12.63	11.30	9.61	10.26	9.73	7.76	8.59	8.84
FOXN3	7.12	6.58	6.57	10.25	9.15	9.36	7.85	8.53	8.03
CNOT1	22.90	22.84	23.09	17.58	17.31	16.60	15.22	15.18	15.44
TOPBP1	8.84	9.93	9.59	8.20	7.76	7.77	6.36	6.66	6.56
HAUS5	4.16	4.82	3.87	4.11	4.01	3.47	2.74	2.93	2.43
PSMA5	13.59	13.67	12.57	14.64	13.80	14.65	12.52	11.52	12.35
CASP2	3.97	3.53	4.11	3.72	3.38	3.68	2.45	2.80	2.86
TMEM14B	22.50	20.96	19.92	17.29	15.47	16.76	14.56	13.79	12.66
MRE11	5.89	5.65	6.26	6.06	5.61	5.99	4.79	4.64	4.81
HAUS8	2.76	2.98	2.46	1.83	2.01	1.66	1.33	1.00	0.98
ANAPC2	8.88	8.37	8.60	8.52	7.94	8.24	6.49	6.97	6.53
DYNLL1	41.15	39.90	38.83	41.61	42.29	43.37	37.37	37.98	36.91
PRMT2	9.97	8.64	9.95	11.60	11.16	11.92	10.12	9.42	10.28
CNOT11	36.28	33.08	34.93	35.07	35.13	35.33	30.93	30.65	30.53
PKIA	7.75	8.08	8.40	9.85	10.29	10.03	8.50	8.27	7.85
CCND1	42.95	44.25	43.97	92.23	92.90	93.26	84.70	87.56	86.72
PSMA7	226.82	205.95	209.13	212.83	206.32	232.90	205.44	192.09	185.99
CTDSPL	5.37	5.27	4.44	4.27	4.68	3.95	2.84	3.17	3.62
JADE1	2.16	2.27	2.15	1.91	1.81	2.20	1.33	1.41	1.54
TCIM	0.06	0.08	0.15	0.32	0.86	1.15	0.14	0.03	0.27
PSMC5	35.28	32.83	37.16	34.99	34.46	36.10	32.61	31.55	30.26
NACC2	10.48	11.65	10.89	10.99	11.02	12.30	10.04	10.05	9.26
FOXO4	1.49	1.07	1.14	2.51	2.25	2.44	1.84	1.46	1.05

SSNA1	60.71	53.55	54.65	57.38	55.53	65.62	51.78	52.02	45.29
CEP70	3.52	4.45	3.45	3.26	3.52	2.85	2.51	2.26	2.14
CDKN1C	15.45	14.26	14.36	21.43	24.70	23.78	18.95	20.49	19.87
CEP41	1.91	1.73	1.60	1.48	1.69	1.52	1.17	1.12	1.30
ANAPC15	4.99	4.03	4.59	5.35	4.43	4.14	3.01	3.64	3.19
ANAPC7	5.53	6.28	5.89	4.73	4.41	3.96	3.33	3.60	3.45
CNOT3	7.15	7.40	7.24	7.05	7.45	6.92	6.22	6.01	6.00
CDC26	7.23	8.02	7.50	8.66	10.01	6.66	5.00	5.59	6.34
PSMD7	19.56	19.61	19.56	17.99	18.58	19.41	16.60	15.81	15.54
CTDSP2	30.41	29.96	31.08	41.94	42.76	42.87	39.67	39.55	39.01
FGFR1OP	1.37	1.56	1.29	0.98	1.03	1.04	0.75	0.80	0.81
CHEK1	9.84	10.37	10.01	10.08	10.68	9.72	8.33	8.85	9.38
PLAGL1	4.53	4.63	4.75	3.62	3.77	3.21	2.63	2.96	3.09
DCTN3	13.90	13.33	12.98	15.84	14.12	16.20	14.10	12.78	13.03
BTG2	6.36	7.32	7.14	9.20	9.22	9.41	8.40	6.99	7.34
PPP2R1A	36.10	33.87	35.86	35.09	33.58	35.64	32.59	32.93	31.81
XRCC3	0.60	0.82	0.86	0.64	0.57	0.60	0.41	0.35	0.37
PSMB10	7.83	7.13	8.01	9.90	11.26	11.14	7.34	10.09	7.94
ADAM17	7.54	8.02	7.96	6.61	6.98	6.64	5.89	6.12	6.11
TPR	13.70	14.57	13.96	12.29	12.55	11.47	10.95	11.11	11.30
CNOT8	9.27	10.90	9.94	12.89	12.61	12.11	11.49	10.23	11.12
RB1	20.35	21.72	22.10	21.89	22.84	21.85	20.25	19.33	21.50
ORC1	4.07	3.90	4.32	3.09	3.69	3.25	2.45	2.76	2.41

Supplementary Table S3. mRNA level of the genes in cell cycle pathway (related to Figure S5C).

SYMBOL	705_Ctrl_1	705_Ctrl_2	705_Ctrl_3	705_Rapamycin_1	705_Rapamycin_2	705_Rapamycin_3	705_RMC-6272_1	705_RMC-6272_2	705_RMC-6272_3
Cdkn1a	21.13	22.10	20.87	7.52	7.08	7.33	1.35	1.87	1.54
Pkmyt1	6.87	6.93	6.79	5.03	4.94	4.84	1.49	1.61	1.45
Gadd45a	4.19	3.96	4.54	45.84	42.28	43.93	5.17	10.70	5.20
Dbf4	12.29	13.14	11.63	10.83	11.72	10.86	4.68	3.67	4.30
Cdc25b	5.88	5.50	6.76	17.09	16.59	16.86	7.37	6.00	6.82
Ccnb1	25.76	27.30	28.19	20.84	20.69	20.45	7.88	5.70	8.17
Espl1	7.29	6.44	7.16	5.53	5.65	5.28	2.25	1.53	2.03
Ccna2	30.93	30.62	30.75	19.31	19.46	20.62	9.44	8.90	9.56
Cdc25c	5.24	5.10	4.85	8.49	8.02	7.04	2.63	2.16	2.84
Mad2l1	8.46	9.06	7.68	5.82	5.56	5.35	2.39	2.70	2.67
Plk1	19.69	21.47	18.32	23.53	21.42	21.24	8.89	6.13	9.72
Cdc45	14.29	15.08	15.71	5.18	5.11	5.37	2.08	2.52	2.13
Bub1	11.57	12.25	11.86	11.77	11.27	11.24	5.07	3.47	5.39
Cdc20	32.78	35.69	37.80	39.85	38.39	41.59	18.79	22.02	18.10
Chek1	6.94	5.55	6.06	3.13	3.07	3.06	1.11	0.73	1.37
Ccnd2	14.91	15.69	15.71	23.95	23.91	24.19	13.34	9.43	13.42
Mcm5	68.74	69.01	69.35	12.52	11.45	11.85	7.26	6.54	6.83
Bub1b	9.99	9.61	8.95	14.77	15.27	14.99	7.71	5.05	8.13
Ccnb2	17.02	17.36	15.85	57.81	51.97	47.57	22.79	31.07	23.55
Ttk	4.76	4.76	4.06	3.65	3.77	4.02	1.93	0.93	1.73
Mcm3	88.36	84.11	89.41	19.18	19.70	19.48	10.83	12.26	12.62
Cdk2	13.43	12.90	13.23	6.98	7.51	7.70	4.76	4.45	4.63
Ccnd1	122.47	120.31	123.53	160.25	157.00	164.63	85.05	47.08	88.83
Ywhag	194.58	200.75	200.51	94.44	93.59	92.84	63.46	48.55	63.99
Orc2	26.63	27.03	25.26	14.37	13.55	13.20	8.84	6.96	9.28
Cdc6	19.97	20.21	20.90	1.41	1.17	1.70	0.72	0.71	0.72
E2f2	1.83	2.43	1.27	0.63	0.84	0.90	0.12	0.41	0.22
Skp2	9.14	9.25	9.41	2.40	2.63	2.82	1.59	1.03	1.68
Cdc7	9.29	9.41	8.70	3.53	3.08	3.72	1.81	0.89	2.21
